# Supplementary material for: De novo active sites for resurrected Precambrian enzymes
Source: Nat Commun. 2017 Jul 18;8:16113. doi: 10.1038/ncomms16113 (PMC5520109; doi:10.1038/ncomms16113)
Supplement: Supplementary Information [file ncomms16113-s1.pdf]

SI GUIDE

File Name: Supplementary Information
Description: Supplementary Figures, Supplementary Tables, Supplementary Notes, Supplementary Methods and Supplementary References.

Supplementary Data

File Name: Supplementary Data 1
Description: Backbone 1H and 15N assignments (ppm) of GNCAMP lactamase.

File Name: Peer Review File
Description:

SUPPLEMENTARY FIGURES

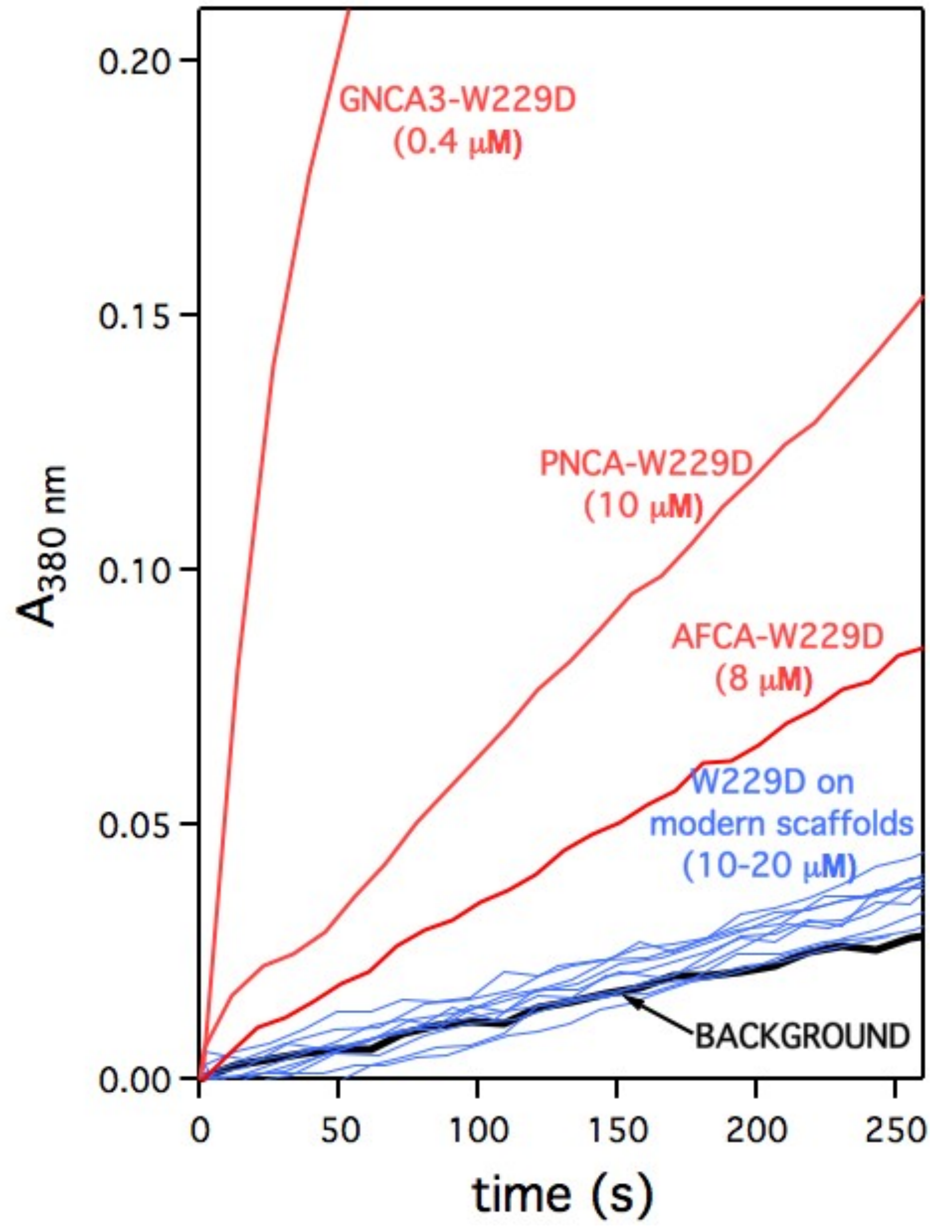

Supplementary Figure 1. Catalytic Activity of Modern and Ancestral β-Lactamases. Shown here are representative examples of the experimental profiles for the catalysis of Kemp elimination by the W229D variants of modern and ancestral β-lactamases. Plotted here are the changes in absorbance at 380 nm (wavelength for the absorption maximum of the product of Kemp elimination) with respect to the initial value *versus* time. All experiments reported here were carried out with a substrate concentration of 500 μM. Profiles for all 10 modern lactamases studied in this work are shown in blue. These were obtained with different protein concentrations within the 10-20 μM range. Note that the profiles are barely distinguishable from the background (enzyme-free) profile shown in black. Profiles for three ancestral lactamases are shown in red. For illustration, we have selected two ancestral scaffolds that lead to a comparatively low Kemp elimination activity (PNCA and AFCA: see Supplementary Table 6) and one ancestral scaffold that leads to a high Kemp elimination activity (GNCA**3**).

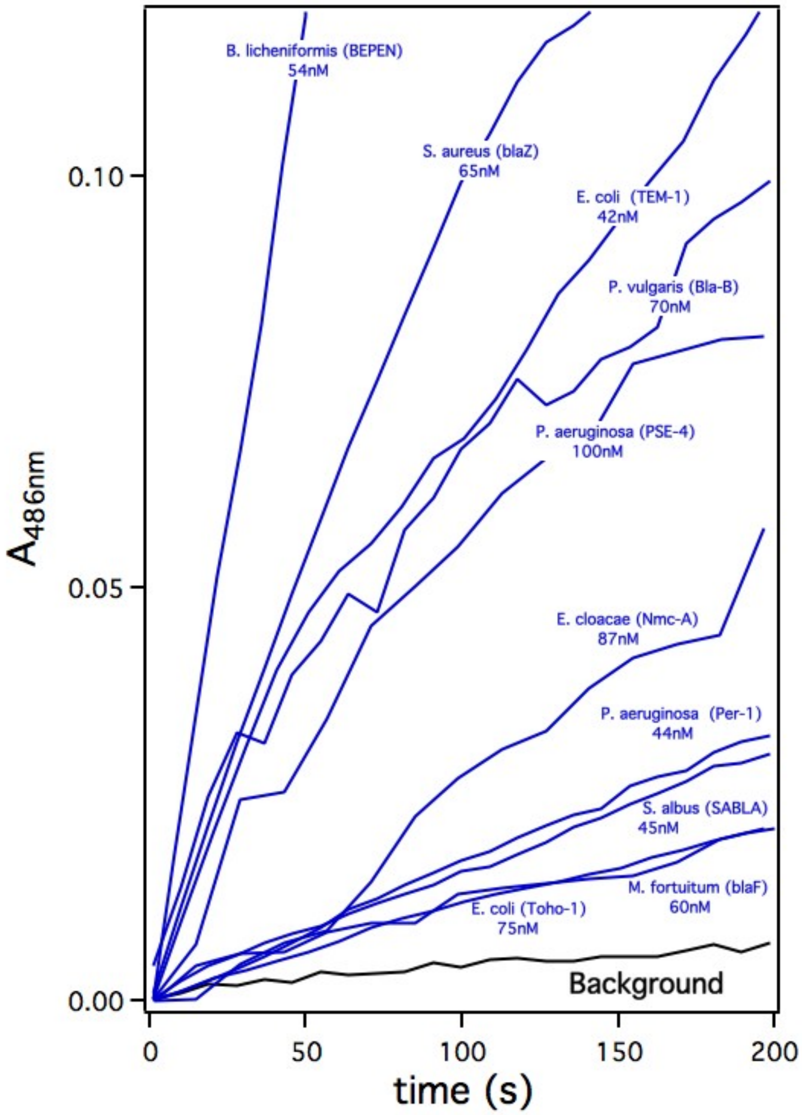

Supplementary Figure 2. Antibiotic Degradation Activity of Modern β-Lactamases
Shown here is the antibiotic degradation activity of the W229D variants of 10 modern β-lactamases. The degradation of nitrocefin is followed by measuring absorbance at 486 nm as a function of time. Nitrocefin is an analog of penicillin with a chromophore attached that allows degradation to be followed by absorbance measurements in the visible region of the spectrum. Note that all W229D variants of the modern lactamases studied here show antibiotic degradation activity at nM concentrations.

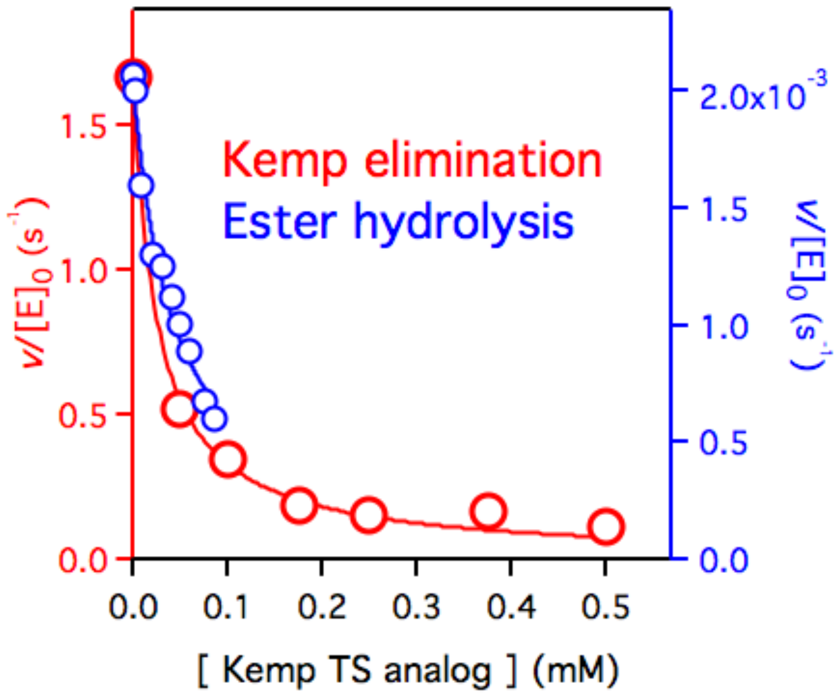

Supplementary Figure 3. Inhibition of the GNCA4-W229D/F290W β-Lactamase

Shown here is the inhibition of the Kemp elimination and ester hydrolysis activities of GNCA4-W229D/F290W by 5(6)-nitrobenzotriazole, which is an analog of the transition state for the Kemp elimination. Ester hydrolysis data were not determined at analog concentrations above about 0.1 mM because of significant catalysis of the reaction by the analog, which leads to substantial hydrolysis in the blanks. Continuous lines represent the best fit of Equation 2 in the main text (based on the Michaelis-Menten mechanism with competitive reversible inhibition). The values of the dissociation constants from these fits are 17.8±1.4 μM and 15.8±1.4 μM for the inhibition of Kemp elimination and *p*-nitrophenyl acetate hydrolysis, respectively [the errors given are the associated standard deviations of the fitting parameters as provided by the fitting program used, Igor Pro 6.37].

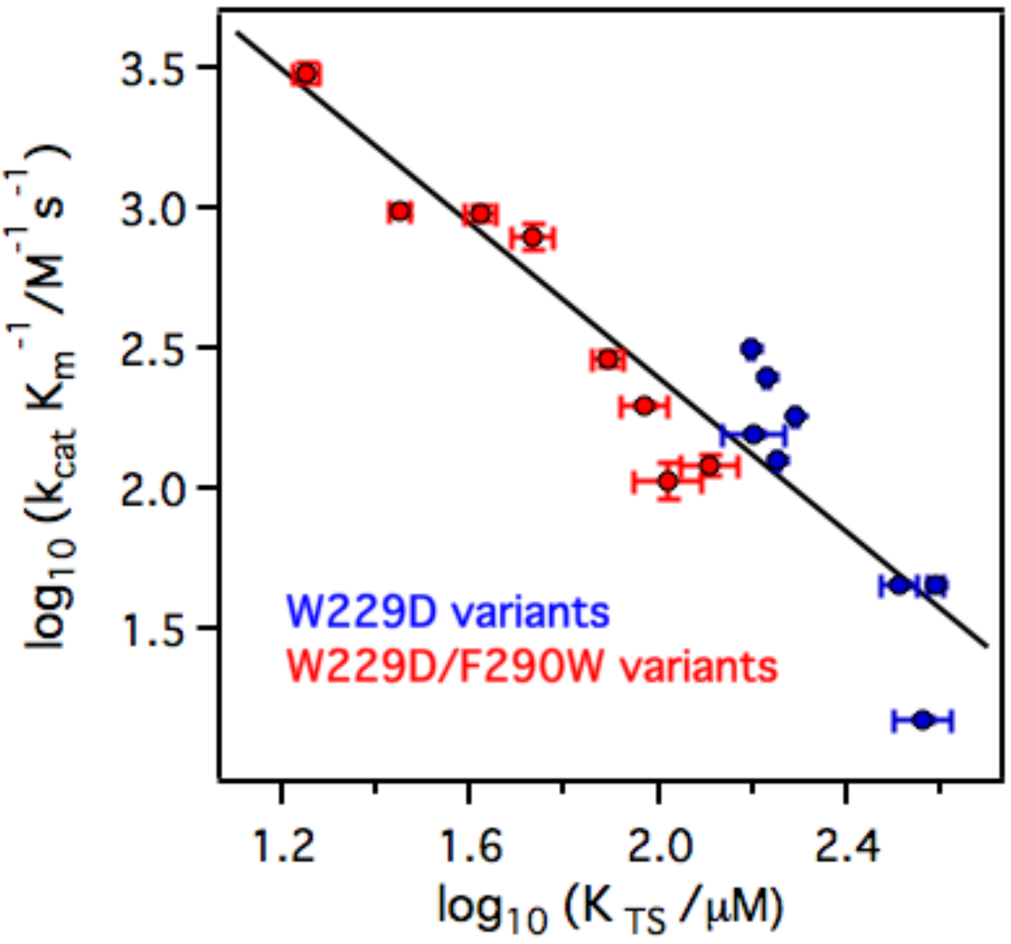

Supplementary Figure 4. Correlation Between Binding and Catalytic Activity
Shown here is the correlation between the *de novo* catalysis of Kemp elimination in ancestral GNCA scaffolds with the binding to the transition-state analog 5(6)-nitrobenzotriazole. K_{TS} is the dissociation constant for the transition-state analog as determined from the experiments on the inhibition of Kemp elimination, such as those shown in Supplementary Figure 3.

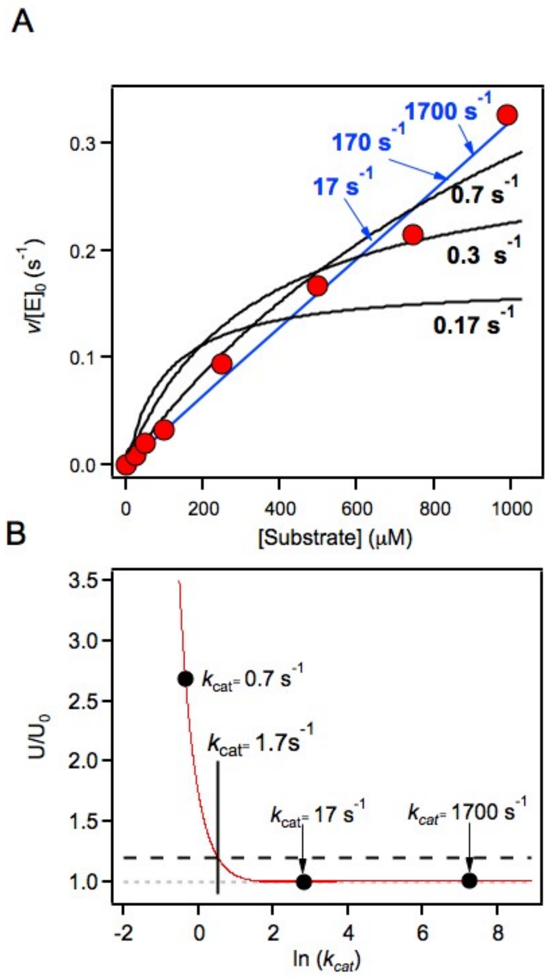

Supplementary Figure 5. Estimation of Lower Limits for the Turnover Number

Many of the plots of rate of Kemp elimination *vs.* substrate concentration determined in this work do not show discernible curvature within the experimentally available substrate concentration range. Therefore, only the catalytic efficiency, k_{cat}/K_M can be calculated from the experimental data as the slope of the linear rate *vs.* [substrate] dependence, while the individual value of k_{cat} is not available. Still, it is possible to place a lower bound on k_{cat} because a linear rate v*s*. [substrate] dependence implies that k_{cat} must be sufficiently above the highest value for the rate/$[E]_0$ determined, since, otherwise, curvature would be observed in the rate *versus* [substrate] plots. This is illustrated in panel A with data corresponding to the GNCA**3**-W229D lactamase. In order to perform the lower-bound estimate in a systematic manner, we resorted to the Bevington method for error estimation[1]. Fits of the Michaelis-Menten equation to each set of data are performed fixing the value of k_{cat} and allowing K_M to vary to optimize the fit. If the value chosen for the k_{cat} is "too low" a poor fit is obtained (fits shown in black in panel A) with a high value for the sum of squares of the deviations (U). If the value of the parameter is sufficiently high, the Michaelis-Menten equation predicts a linear dependence within the experimental substrate concentration range (fits shown in blue in panel A) and a good fit with the "best" value of U (U_0) is obtained. According to Bevington, the lower limit estimate of k_{cat} easily determined as the value of the parameter for which U/U_0 equals N-n+1/(N-n), where N is the number of experimental data points and n is the number of fitting parameters. In this case, this value is 1.7 s^{-1}. This means that, while we do not know the exact value of k_{cat}, values smaller than 1.7 s^{-1} are not realistic for GNCA**3**-W229D (because they would imply a curvature in the Michaelis plot that is inconsistent with the experimental data).

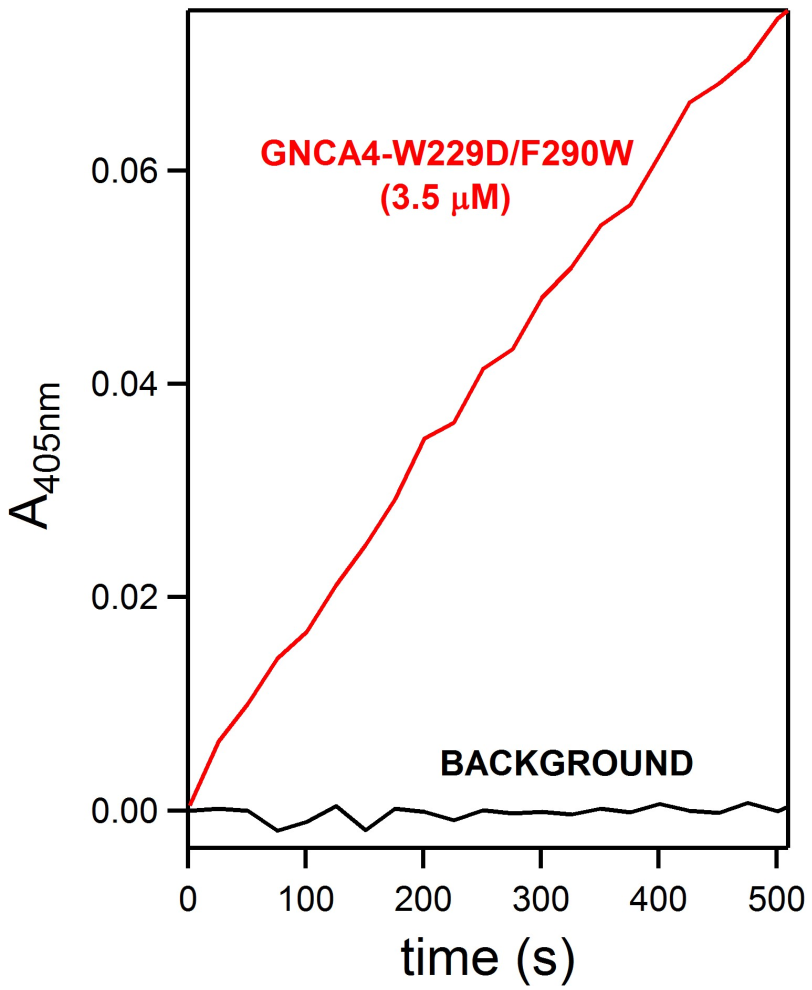

Supplementary Figure 6. Catalysis of *p*-Nitrophenyl Acetate by GNCA4-W229D/F290W
Shown here is an example of the experimental profile for the catalysis of *p*-nitrophenyl acetate by GNCA4-W229D/F290W. Changes in absorbance at 405 nm (a wavelength at which the product of the reaction, *p*-nitrophenol, shows strong absorption) with respect to the initial value are plotted *versus* time. The experiment reported here was carried out with a substrate concentration of 300 μM. At this concentration the esterase activity of GNCA4-W229D/F290W is dominated by the new active site (see Figure 7 in the main text). The profile for the background (enzyme-free) reaction is shown in black.

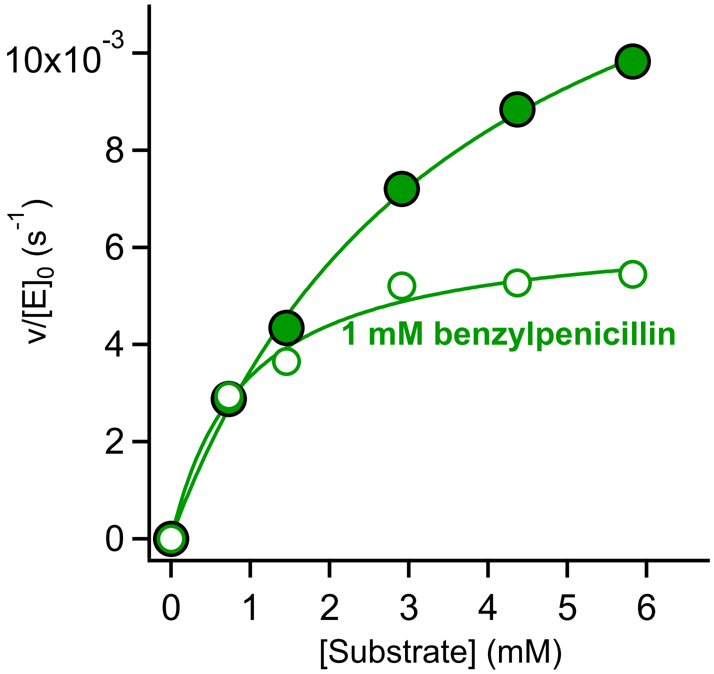

Supplementary Figure 7. Esterase Activity of the GNCA4-W229D/F290W β-Lactamase
Shown here is a plot of the rate (v/[E]$_0$) *vs.* substrate (*p*-nitrophenyl acetate) concentration for the esterase activity of the W229D/F290W variant of the ancestral GNCA4 scaffold. Experiments were carried our according to the following protocol: for each substrate concentration, hydrolysis was followed spectrophotometrically for a time sufficient to allow an accurate determination of the rate. Subsequently, 1 mM benzylpenicillin was added and the rate was measured again. This antibiotic concentration is much larger than the K_M value and, therefore, the natural active site is expected to be saturated by the antibiotic despite its hydrolysis. The rates in the absence and presence of benzylpenicillin are shown with closed and open symbols, respectively. The results shown here are in agreement with those obtained by blocking the natural active by mutation and by inactivation with clavulanic acid (see Figure 7 in the main text).

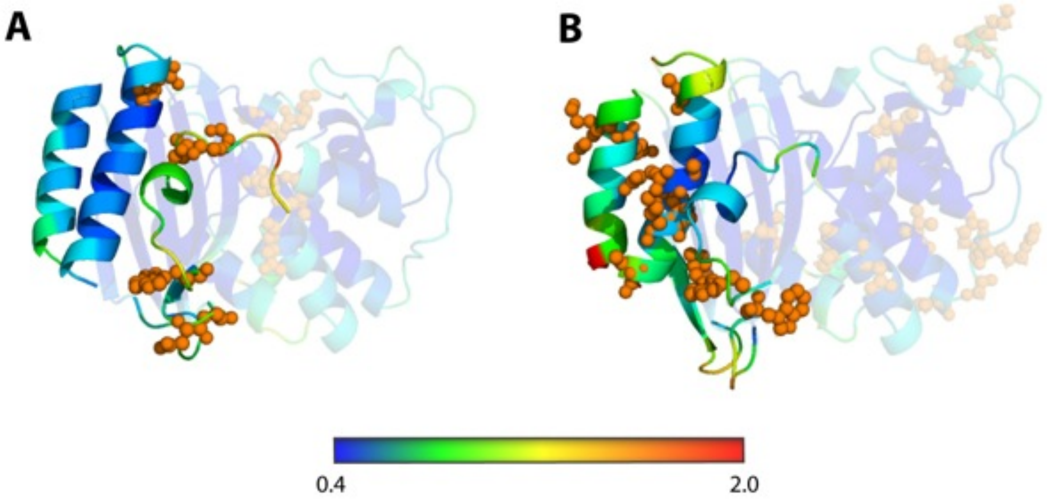

Supplementary Figure 8. Structural Comparison Between TEM1 and GNCA$_{MP}$
Shown here are the tertiary structures of the TEM-1 and GNCA$_{MP}$ β-lactamases with the region of the *de novo* active site and the residues with exchange contributions to the NMR relaxation highlighted. The protein backbone is colored according to the C_{α} atom root mean square fluctuation (RMSF) values calculated from the MD simulations, and it can be seen that the residues with exchange contributions to the NMR relaxation match the elevated RMSF at the *de novo* active-site region. The color scale here is the same as that used in Figure 9 of the main text. Residues with exchange contributions to the NMR relaxation rates are highlighted by displaying their side-chains in orange.

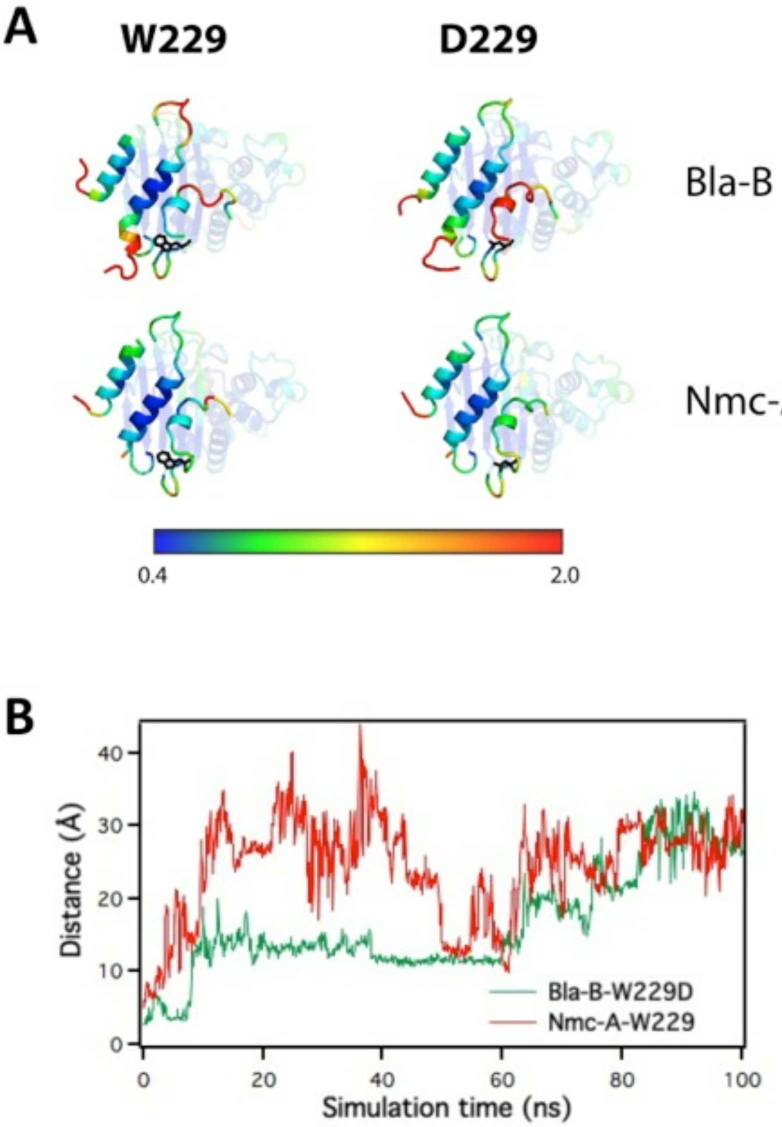

Supplementary Figure 9. Flexibility of β-lactamases from *E. Cloacea* and *P. Vulgaris*
Shown here are results of molecular dynamics (MD) simulations with modern β-lactamases from *P. vulgaris* (Bla-B) and *E. cloacae* (Nmc-A). (A) Tertiary structures of the wild-type and W229D variants colored by the C_α atom root mean square fluctuation (RMSF) value (see Figure 9 in the main text for comparison). (B) Distance (in Å) between the nitrogen atom of the 5(6)-nitrobenzotriazole TSA and the closest atom of D229 in simulations of the enzyme-TSA complexes. These simulations are equivalent to those shown in Figure 10 of the main text. The large values reached for the distance after 100 ns of simulation time indicate that, as with the other modern enzymes studied in this work, the proteins cannot retain the TSA in the pocket generated by the W229D mutation.

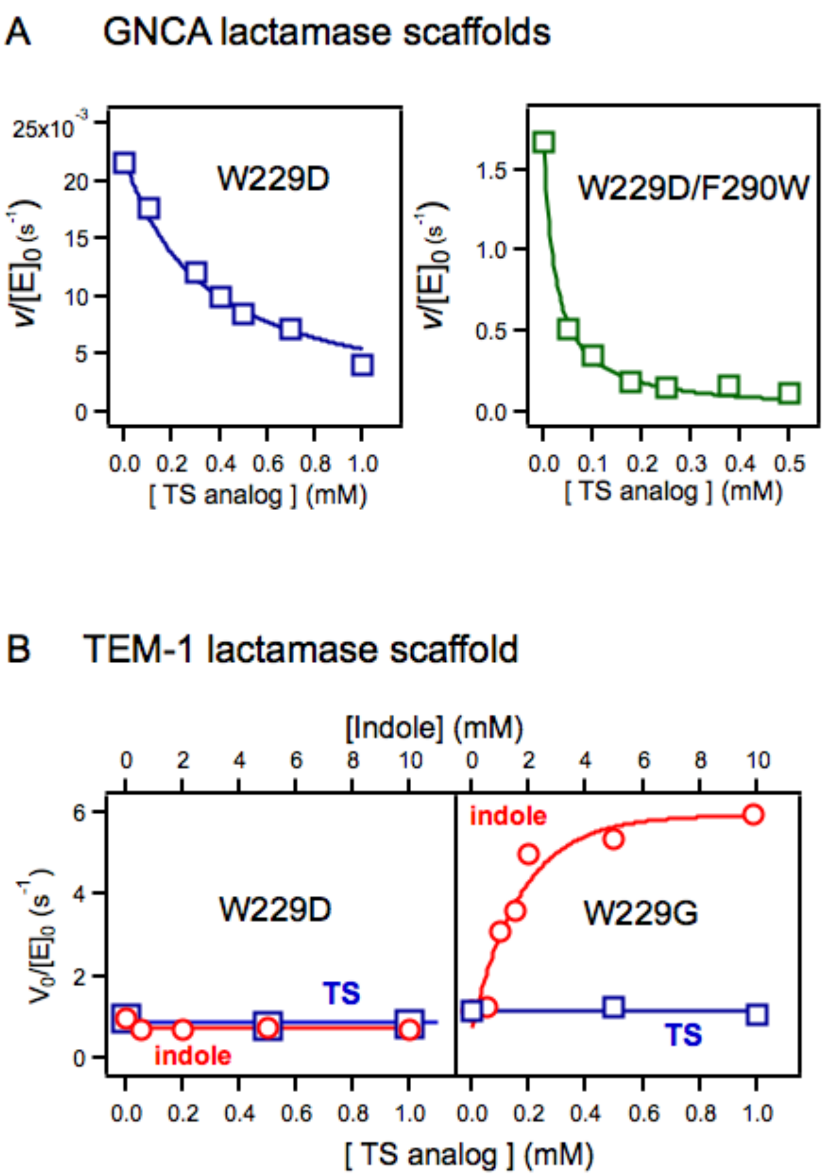

Supplementary Figure 10. Ligand Binding to Ancestral and Modern β-Lactamases
Shown here is the binding of indole and the transition-state analog 5(6)-nitrobenzotriazole (TSA) to ancestral and modern β-lactamase scaffolds with cavity-creating mutations at position 229. A) Binding to GNCA$_{MP}$-W229D (left) and GNCA4-W229D/F290W (right) β-lactamases followed by inhibition of the Kemp elimination. The continuous lines are the best fits of equation 2 in the main text (based on the Michaelis-Menten mechanism with competitive reversible inhibition). B) Binding to variants of the modern TEM-1 β-lactamase with the W229D (left) and W229G (right) mutations followed by the rescue of the antibiotic (nitrofecin) degradation activity; in both cases, the global suppressor mutation M182T was included for stabilization. Note that there is no binding of the transition state analog or even indole to the TEM1-W229D/M182T variant. On the other hand, the indole does bind to the W229G variant of the TEM-1 scaffold, although not to the W229D variant (which, in both cases, were previously stabilized by the global suppressor M182T mutation). It appears, therefore, that binding to the modern TEM-1 lactamase is possible, but only when the shape of the ligand matches closely the shape of the cavity generated at position 229 (in a rigid molecular environment, a W229G mutation should generate a cavity of the shape of indole, the tryptophan side chain).

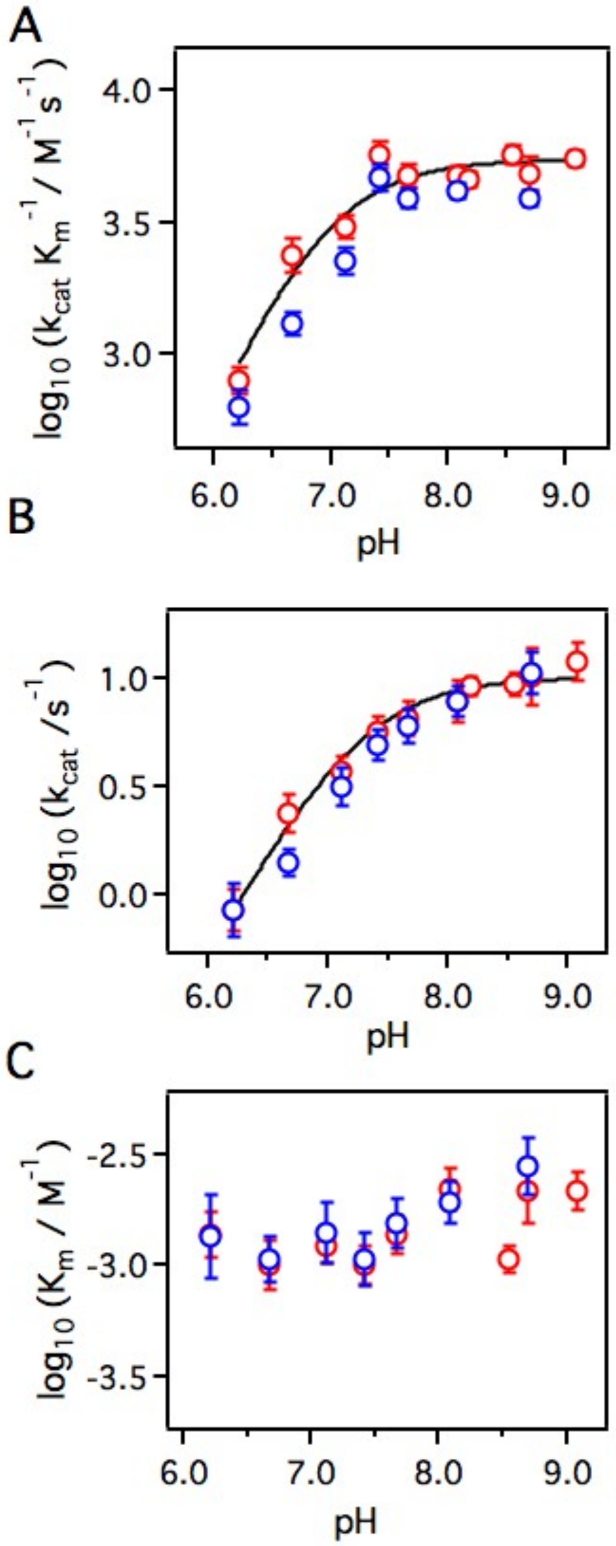

Supplementary Figure 11. pH-dependence of the GNCA4-W229D/F290W β-Lactamase
Shown here is the pH dependence of the catalytic parameters for GNCA4-W229D/F290W Kemp eliminase. A) Plot of catalytic efficiency (k_{cat}/K_M) versus pH. B) Plot of turnover number (k_{cat}) versus pH. C) Plot of Michaelis constant (K_M) versus pH. The colors refer to the presence (red) or absence (blue) of a His-tag.

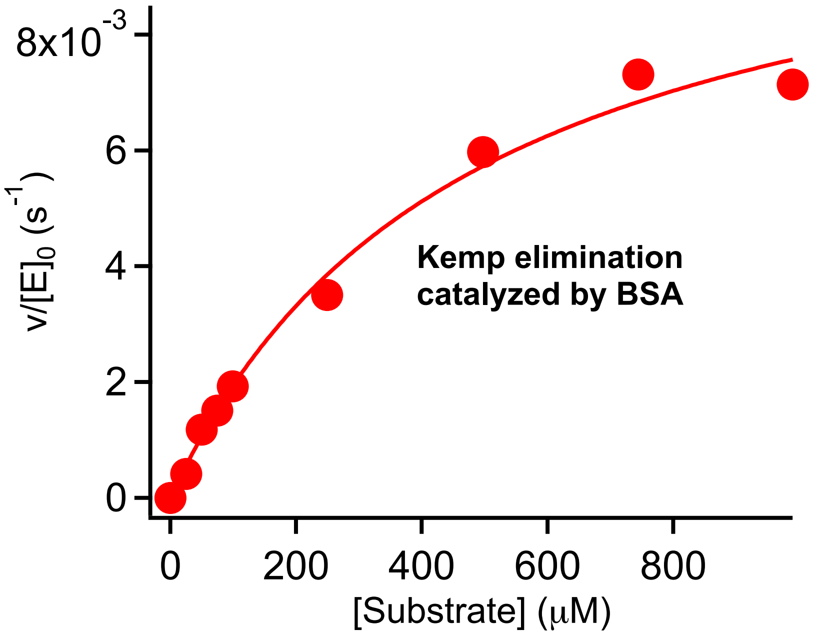

Supplementary Figure 12. Catalysis of Kemp Elimination by Bovine Serum Albumin
Profiles of rate ($v/[E]_0$) vs. substrate concentration. Experiments were performed with a BSA concentration of 11.5 μM at 25 ºC in 50 mM HEPES (4-(2-hydroxyethyl)-1-piperazinesulfonic acid) buffer pH 7. The continuous line is the best fit of the Michaelis-Menten to the experimental data. The catalytic parameters derived from this fit (k_{cat}=0.011±0.001 s^{-1} and K_m=475±90 μM) are in good agreement with those reported by Hilvert and coworkers[2] under slightly different solvent conditions: k_{cat}=0.017±0.001 s^{-1} and K_m=720±68 μM in 10 mM phosphate buffer, 100 mM NaCl pH 7.4, 20 ºC.

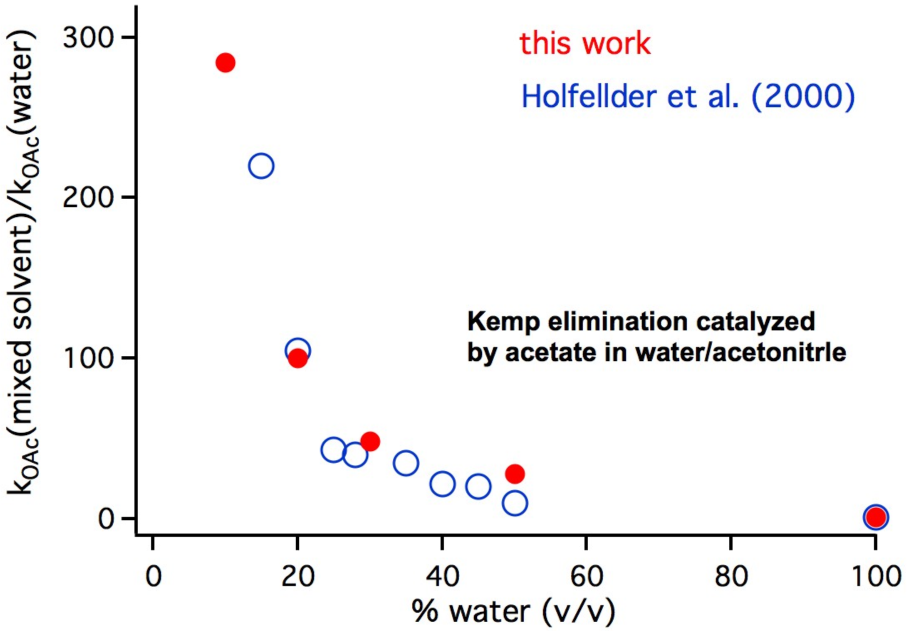

Supplementary Figure 13. Kemp Elimination by Acetate Ion in Mixed Solvents
Shown here are plots for Kemp elimination by acetate ion in water/acetonitrile mixtures. The mixed solvents were generated by mixing 100 mM HEPES buffer pH 7 with acetonitrile. Sodium acetate was included and the catalytic constants for the acetate ion were derive from the comparison of the rates obtained in the presence and absence of acetate. Data obtained in this work are shown with red closed data symbols. Open blue symbols represent the values reported by Hollfelder *et al*.[3]

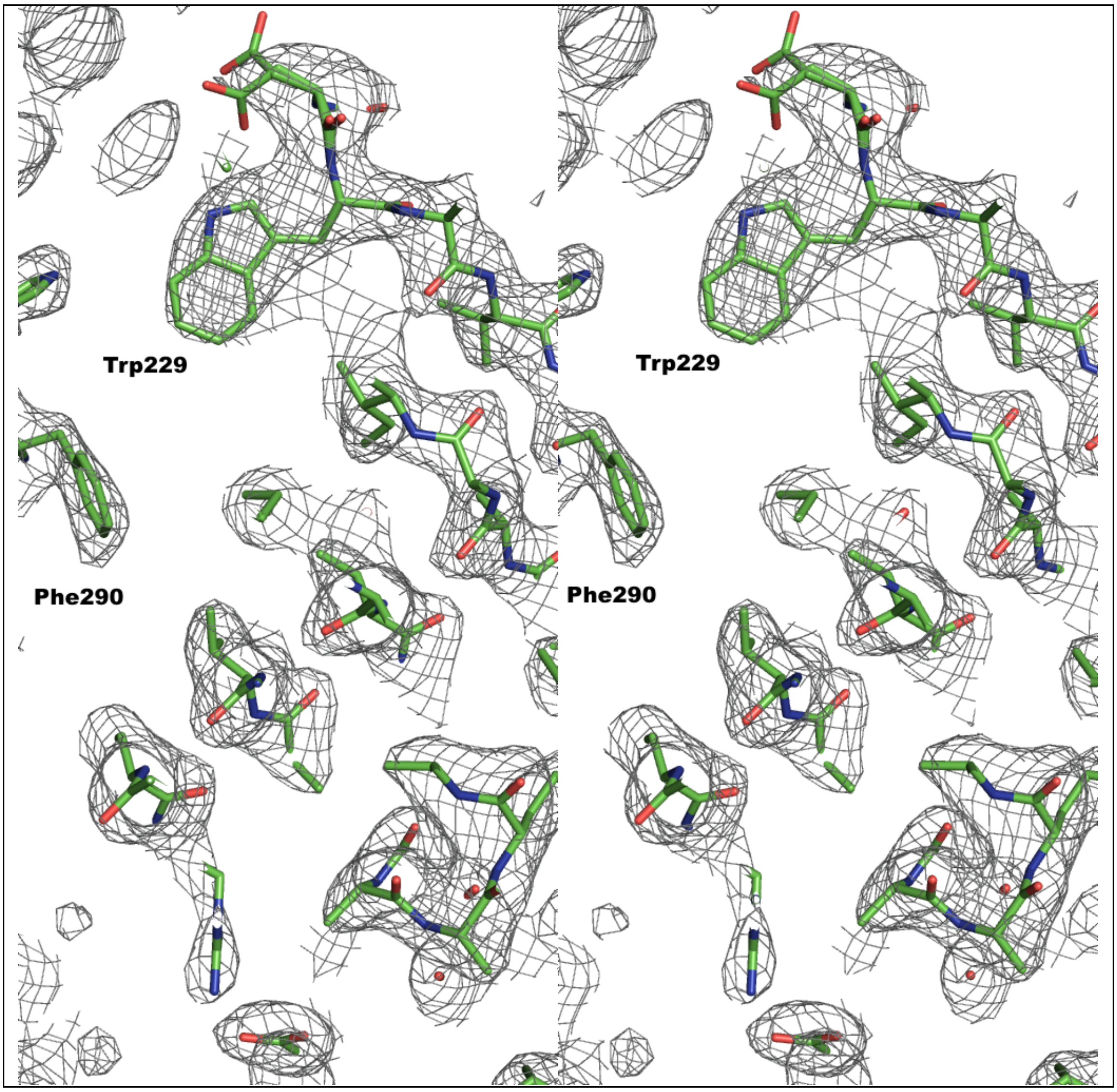

Supplementary Figure 14. GNCA4 electron density map.
Stereo view of a relevant portion of the GNCA4 3D model with the |2Fo-Fc| electron density map at 2.12 Å resolution contoured at 1.0 σ (grey mesh). Some relevant amino acids are labelled.

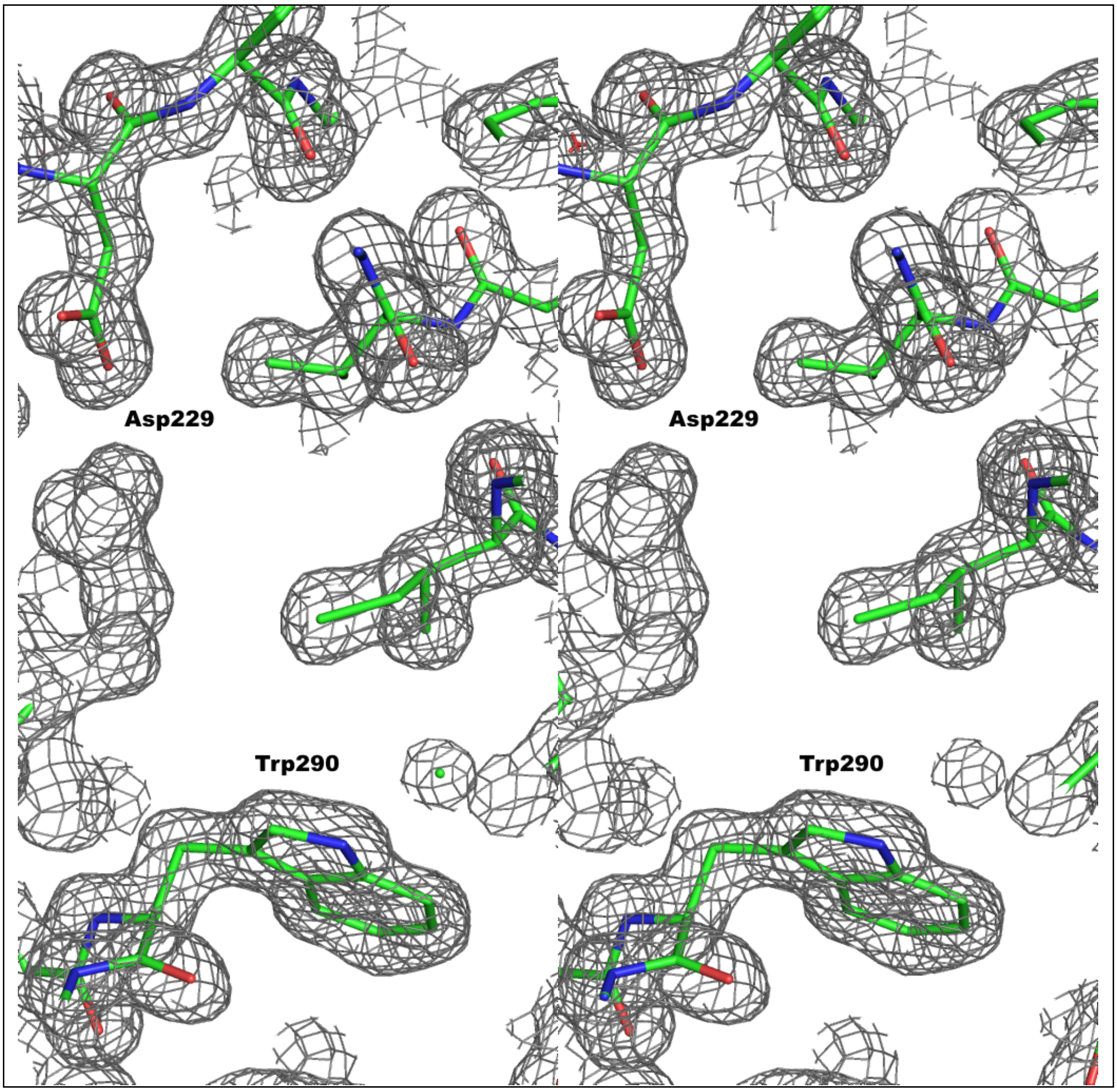

Supplementary Figure 15. GNCA4 W229D/F290W electron density map.
Stereo view of a relevant portion of the GNCA4 W229D/F290W GNCA4 model with the |2Fo-Fc| electron density map at 1.4 Å resolution contoured at 1.0 σ (grey mesh). Some relevant amino acids are labelled.

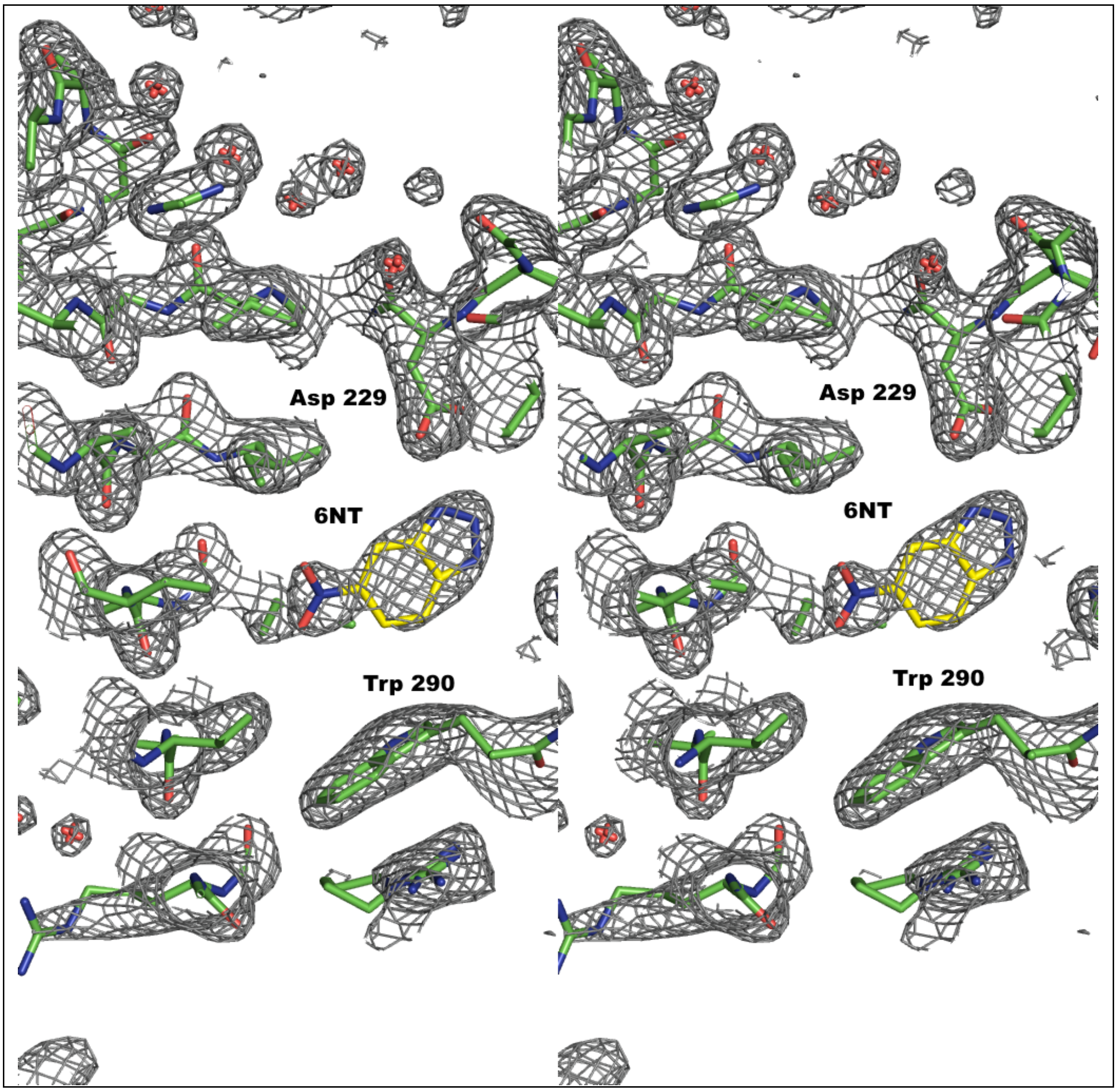

Supplementary Figure 16. GNCA4 W229D/F290W electron density map with TSA.
Stereo view of a relevant portion of the GNCA4 W229D/F290W 3D model with the |2Fo-Fc| electron density map at 1.77 Å resolution contoured at 1.0 σ (grey mesh). Some relevant amino acids and the bound transition-state analogue (6NT) are labelled.

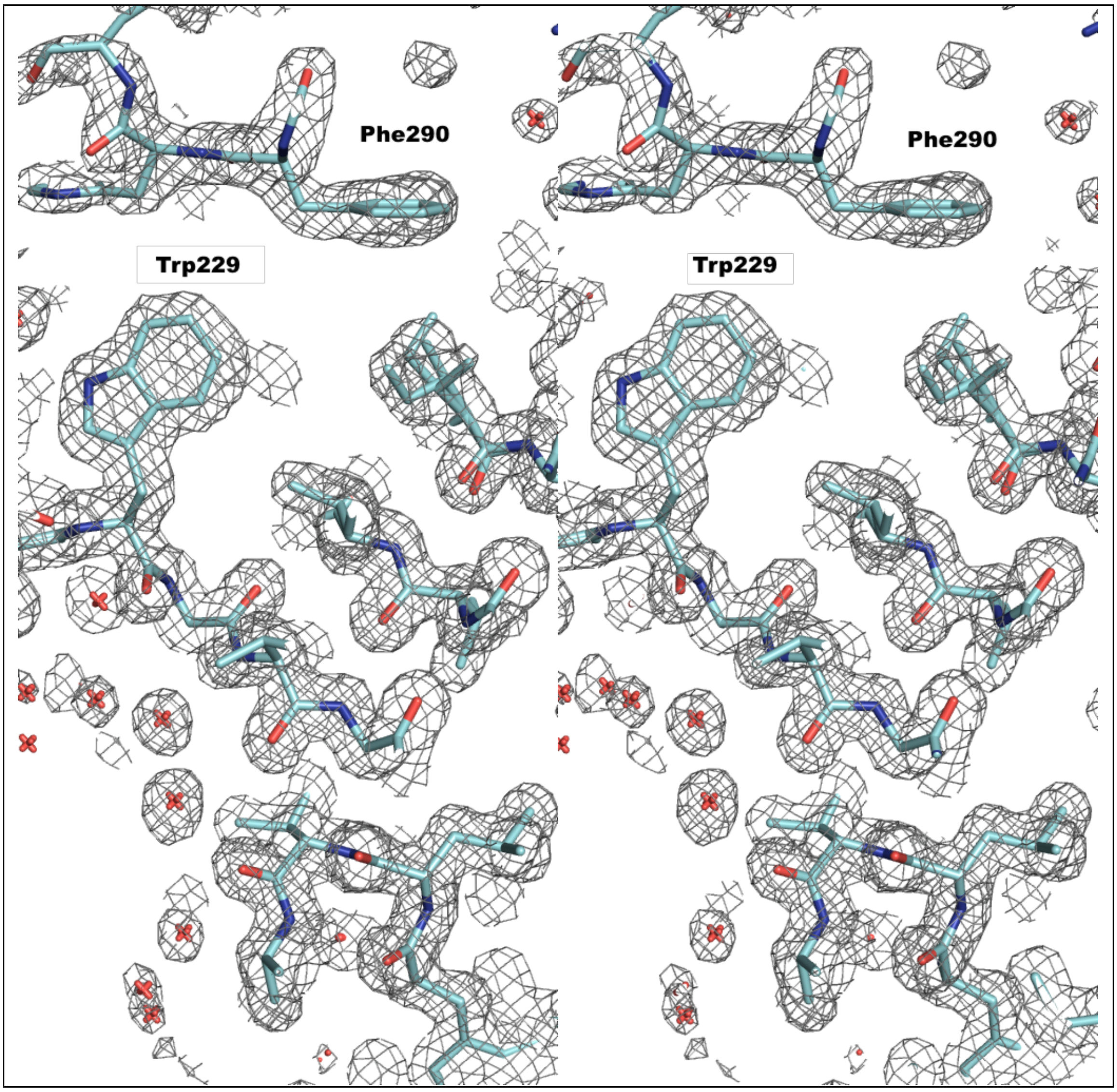

Supplementary Figure 17. GNCA$_{MP}$ electron density map.
Stereo view of a relevant portion of the GNCA$_{MP}$ 3D model with the |2Fo-Fc| electron density map at 1.5 Å resolution contoured at 1.0 σ (grey mesh). Some relevant amino acids are labelled.

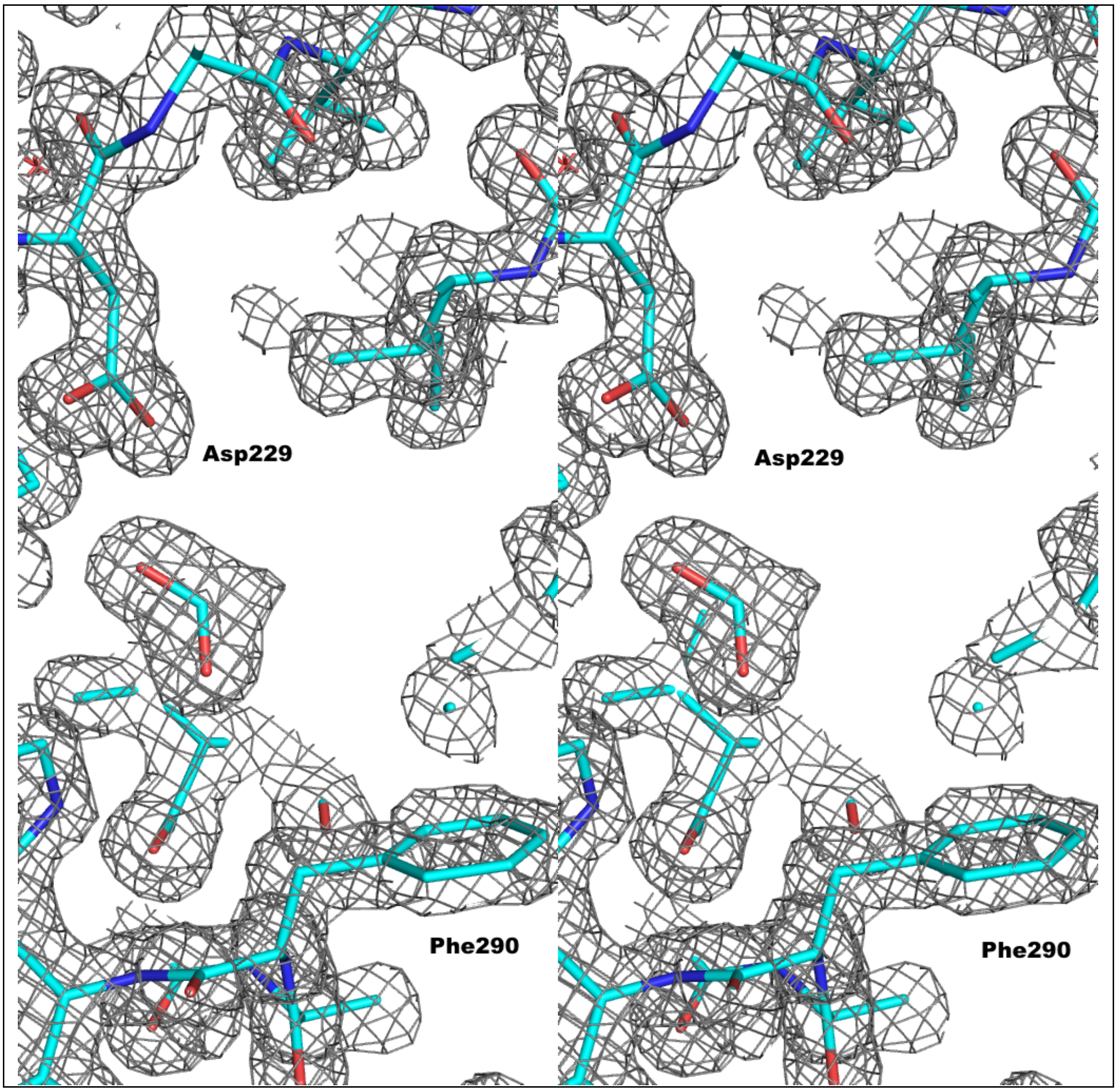

Supplementary Figure 18. GNCA$_{MP}$ W229D electron density map.
Stereo view of a relevant portion of the GNCA$_{MP}$ W229D 3D model with the |2Fo-Fc| electron density map at 1.3 Å resolution contoured at 1.0 σ (grey mesh). Some relevant amino acids are labelled.

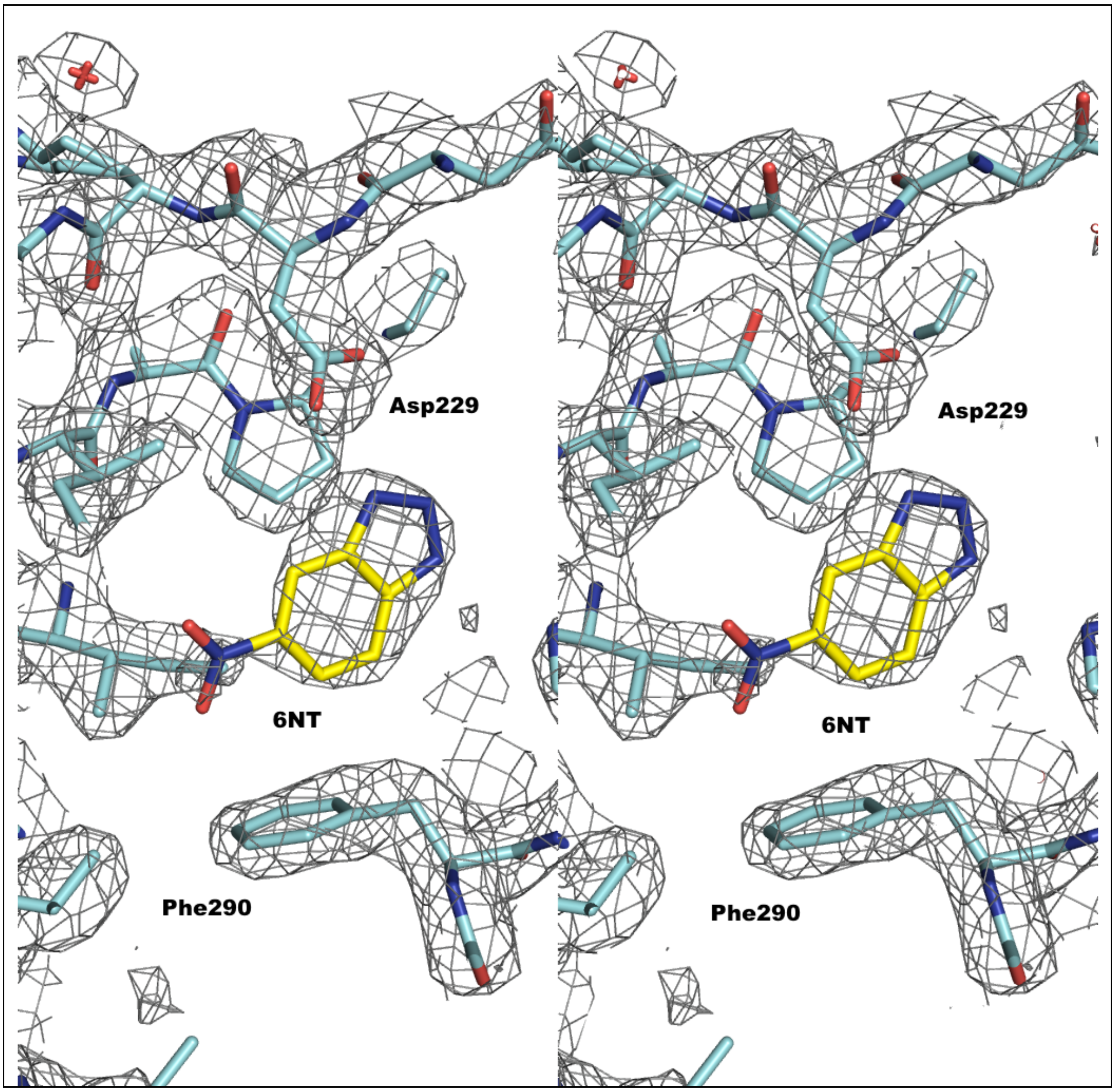

Supplementary Figure 19. GNCA$_{MP}$ W229D electron density map with TSA. Stereo view of a relevant portion of the GNCA4$_{MP}$ W229D 3D model with the |2Fo-Fc| electron density map at 2.27 Å resolution contoured at 1.0 σ (grey mesh). Some relevant amino acids and the bound transition-state analogue (6NT) are labelled.

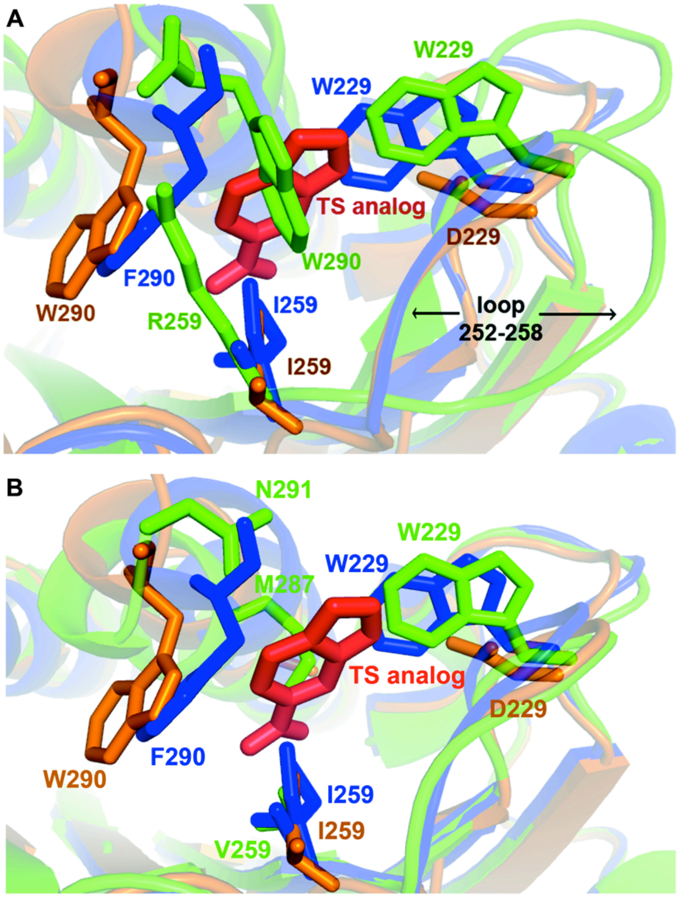

Supplementary Figure 20. Comparison Between Modern and Ancestral β-Lactamases
Shown here is a superposition of the 3D structures for modern and ancestral β-lactamases (see Supplementary Note for details). A) Superposition of the 3D structures for modern TEM-1 β-lactamase (green), GNCA4 β-lactamase (blue) and our best Kemp eliminase, GNCA**4**-W229D/F290W β-lactamase (orange) with the transition-state analog 5(6)-nitrobenzotriazole (red) bound at the de novo active site. B) Superposition of the 3D-structures for modern *Bacillus licheniformis* β-lactamase (green), GNCA**4** β-lactamase (blue) and our best Kemp eliminase, GNCA**4**-W229D/F290W β-lactamase (orange) with the transition-state analog 5(6)-nitrobenzotriazole (red) bound at the de novo active site. In both panels, only the local environment of position 229 is shown.

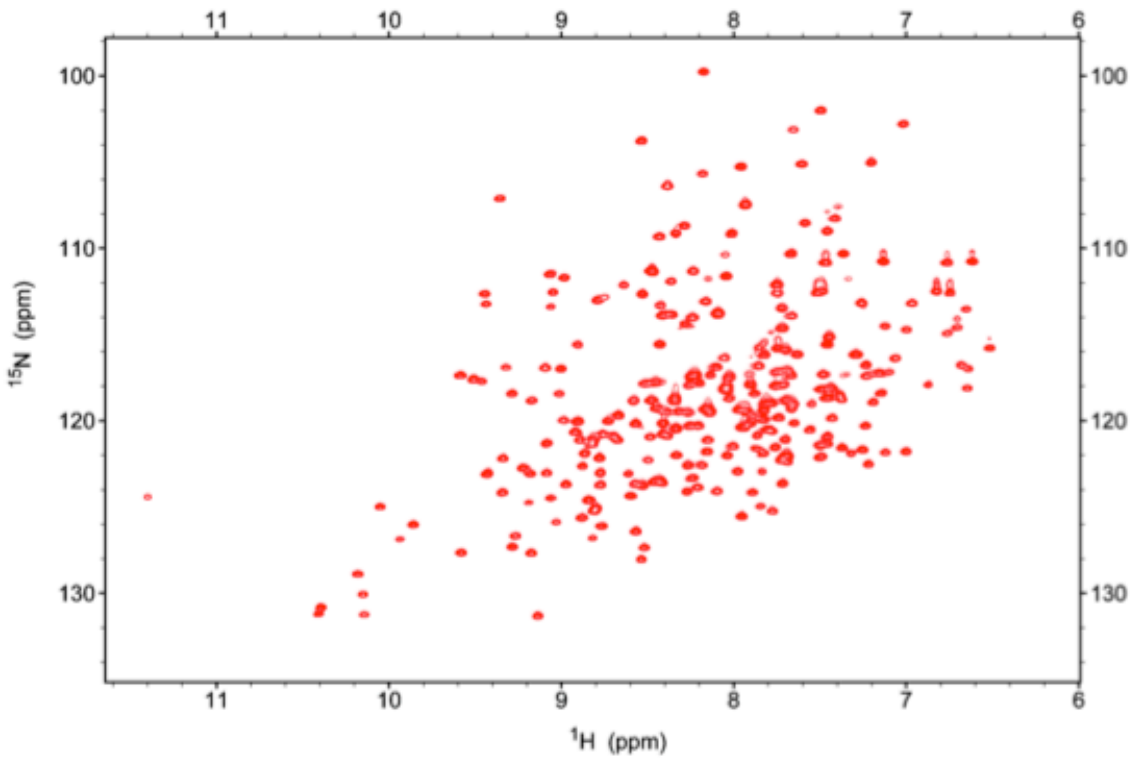

Supplementary Figure 21. ^{1}H-^{15}N HSQC spectrum of the GNCA$_{MP}$ β-lactamase. See Supplementary Methods for details.

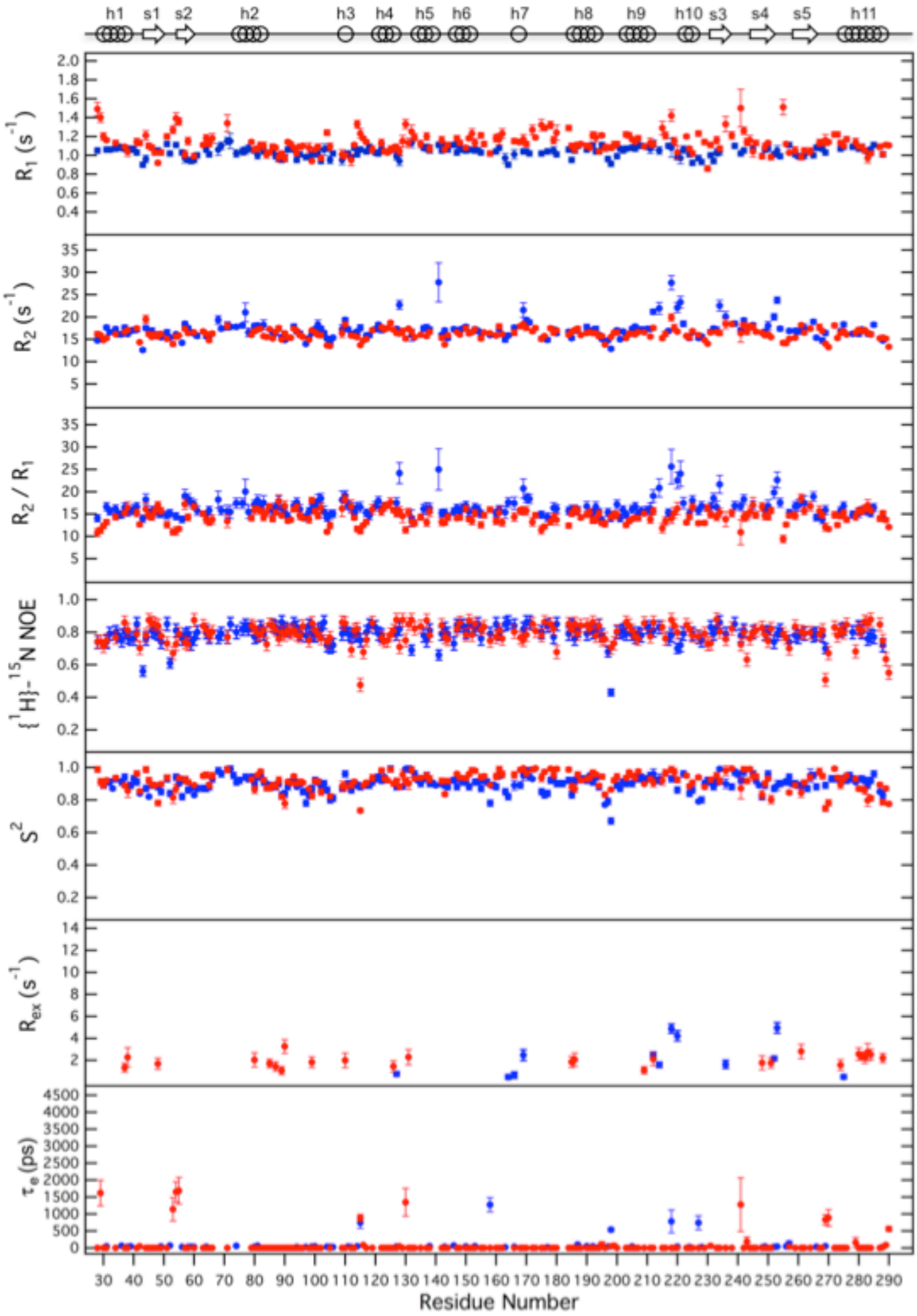

Supplementary Figure 22. Relaxation and Internal Parameters Obtained by NMR
Shown here are data in blue for TEM-1 and in red for $GNCA_{MP}$. The secondary structure is shown above the graph. See Supplementary Methods for details.

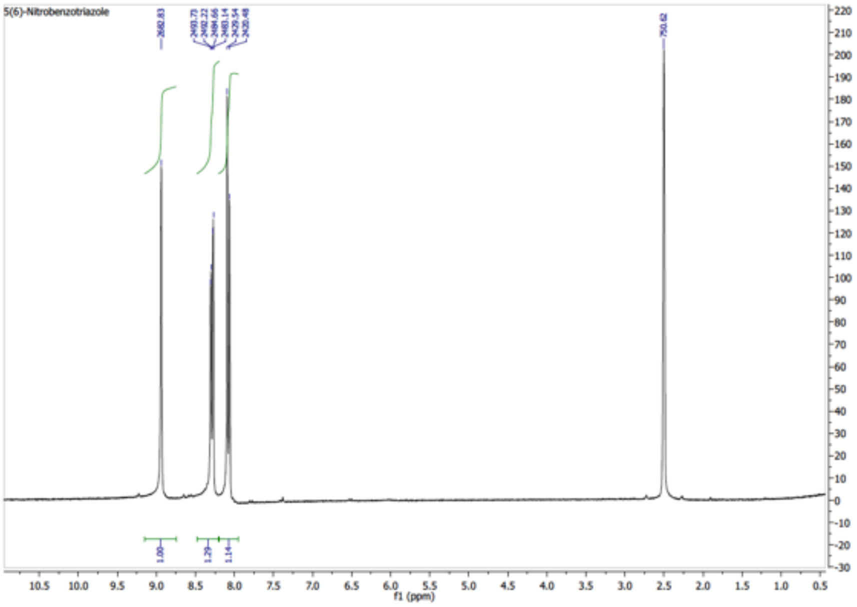

Supplementary Figure 23. 1D-NMR Spectrum of 5(6)-Nitrobenzotriazole

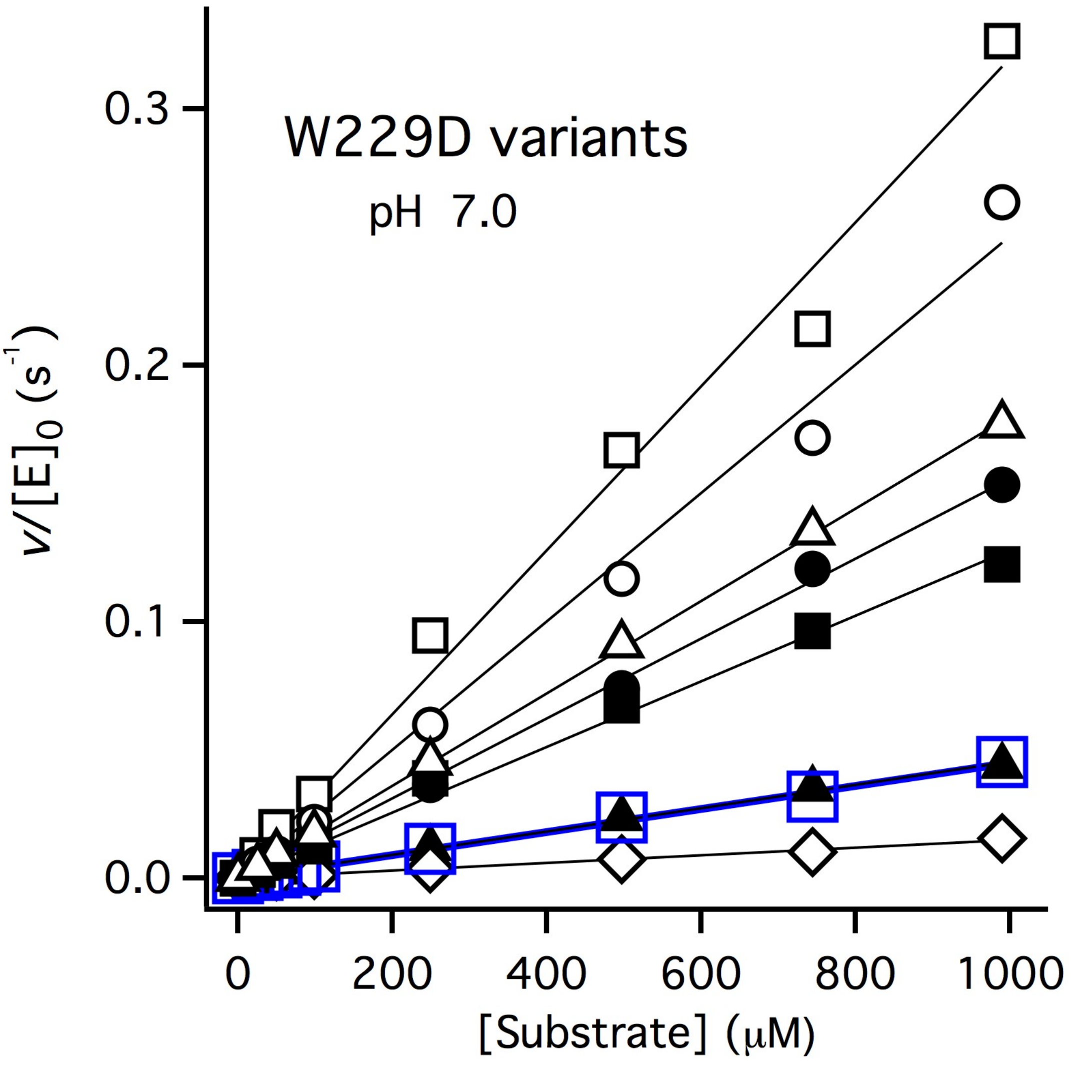

Supplementary Figure 24. Catalysis by W229D Ancestral β-Lactamases
Shown here is the catalysis of Kemp elimination by W229D variants of putative ancestral β-lactamases at pH 7. Profiles of rate ($v/[E]_0$) *vs.* substrate concentration for W229D variants of ancestral GNCA lactamases are shown. The symbols refer to the different representations (Supplementary Table 3) of the lactamase corresponding to the GNCA node: $GNCA_{MP}$ (large blue squares); GNCA1 (closed circles); GNCA2 (closed triangles); GNCA3 (open squares); GNCA4 (open circles); GNCA5 (open triangles); GNCA6 (closed squares); GNCA7 (open diamonds).

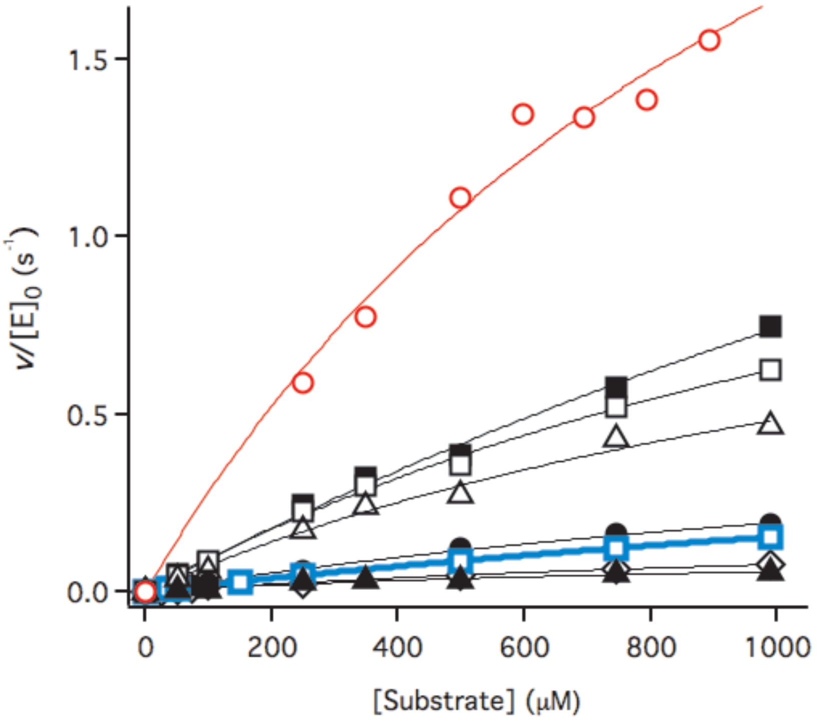

Supplementary Figure 25. Catalysis by W229D/F290W Ancestral β-Lactamases
Shown here is the catalysis of Kemp elimination by W229D/F290W variants of putative ancestral β-lactamases at pH 7. Profiles of rate ($v/[E]_0$) *vs.* substrate concentration for W229D/F290W variants of ancestral GNCA lactamases are shown. The symbols refer to the different representations (Supplementary Table 3) of the lactamase corresponding to the GNCA node: $GNCA_{MP}$ (large blue squares); GNCA1 (closed circles); GNCA2 (closed triangles); GNCA3 (open squares); GNCA4 (red open circles); GNCA5 (open triangles); GNCA6 (closed squares); GNCA7 (open diamonds). The continuous lines represent the best fits of the Michaelis-Menten equation.

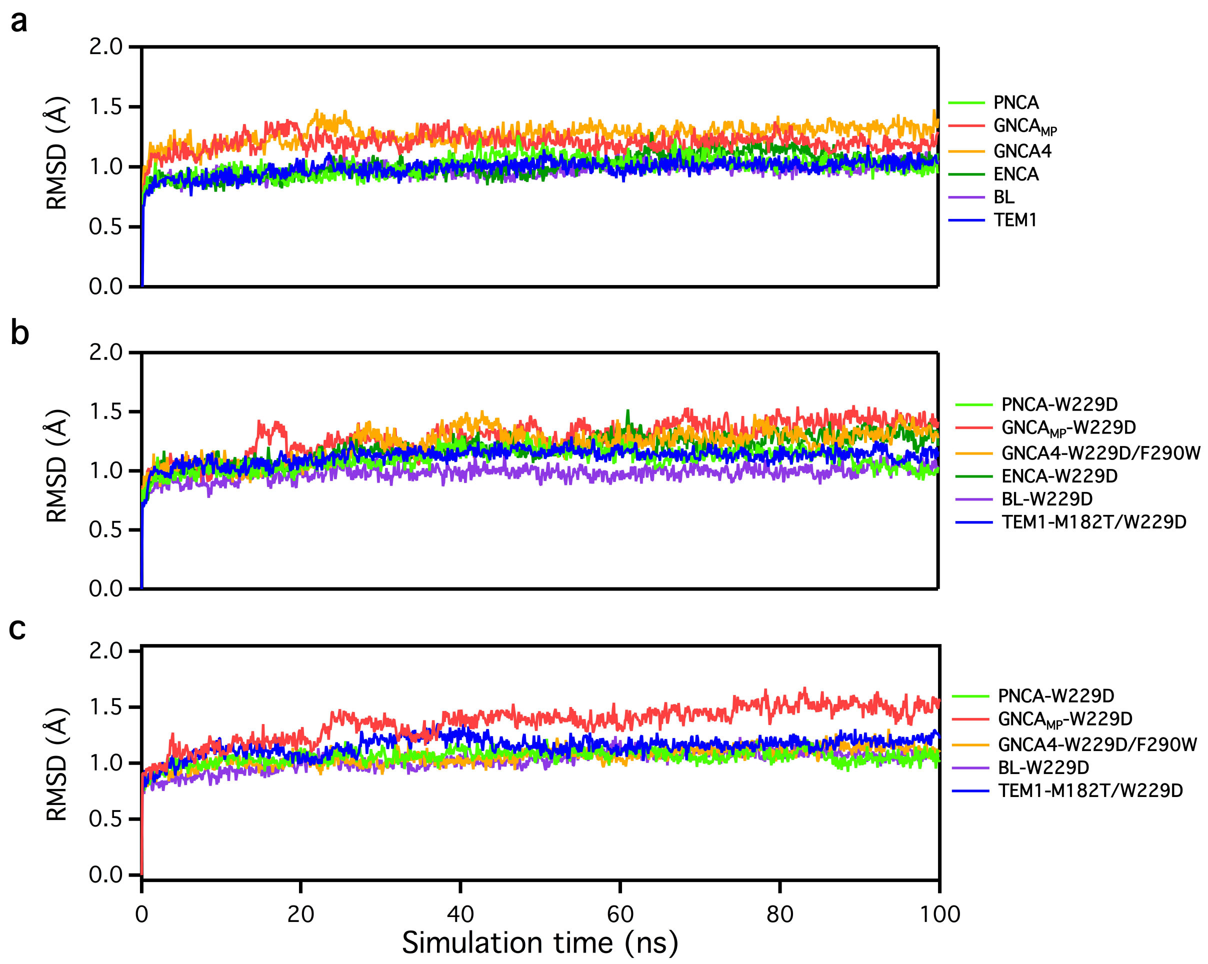

Supplementary Figure 26. C_α Root Mean Square Deviations During Our Simulations
Root mean square deviations (RMSD) in Å of all C_α atoms during the course of our simulations modern and ancestral lactamases: (A) wild-type and (B,C) variant forms of the TEM1 (blue), *Bacillus licheniformis* (purple), $GNCA_{MP}$ (red), PNCA (light green), ENCA (dark green) and GNCA4 (orange) lactamases. In the case of the variant forms, the simulations were performed either in the absence (B) or presence (C) of the transition state analog (TSA) 5(6)-nitrobenzotriazole. All values are averages over three independent trajectories, which were obtained as described in the Methods section of the main text.

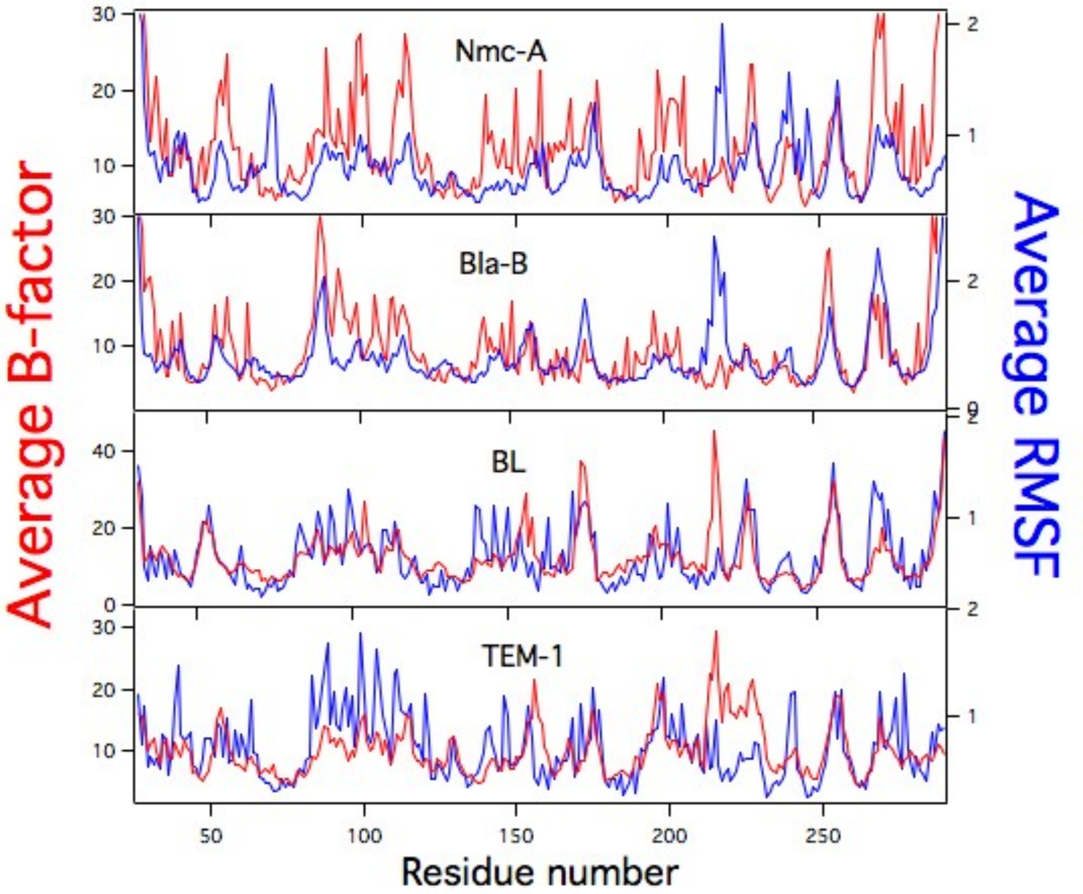

Supplementary Figure 27. Structural Validation of Modern β-Lactamase Simulations
Comparison between root mean square fluctuations and crystallographic B-factors for several modern β-lactamases studied in this work. Shown here are the data for the TEM-1 β-lactamase and for the β-lactamases from *B. licheniformis* (BL), *P. vulgaris* (Bla-B) and *E. cloacae* (Nmc-A). The average residue values for RMSF and B-factor are plotted *vs.* the residue number. The overall congruence observed (regions of high RMSF often, although not always, match regions of high B-factor) appears comparable to that reported by Orozco and co-workers in their consensus analysis of protein dynamics (see Figure 3 in ref. [4]).

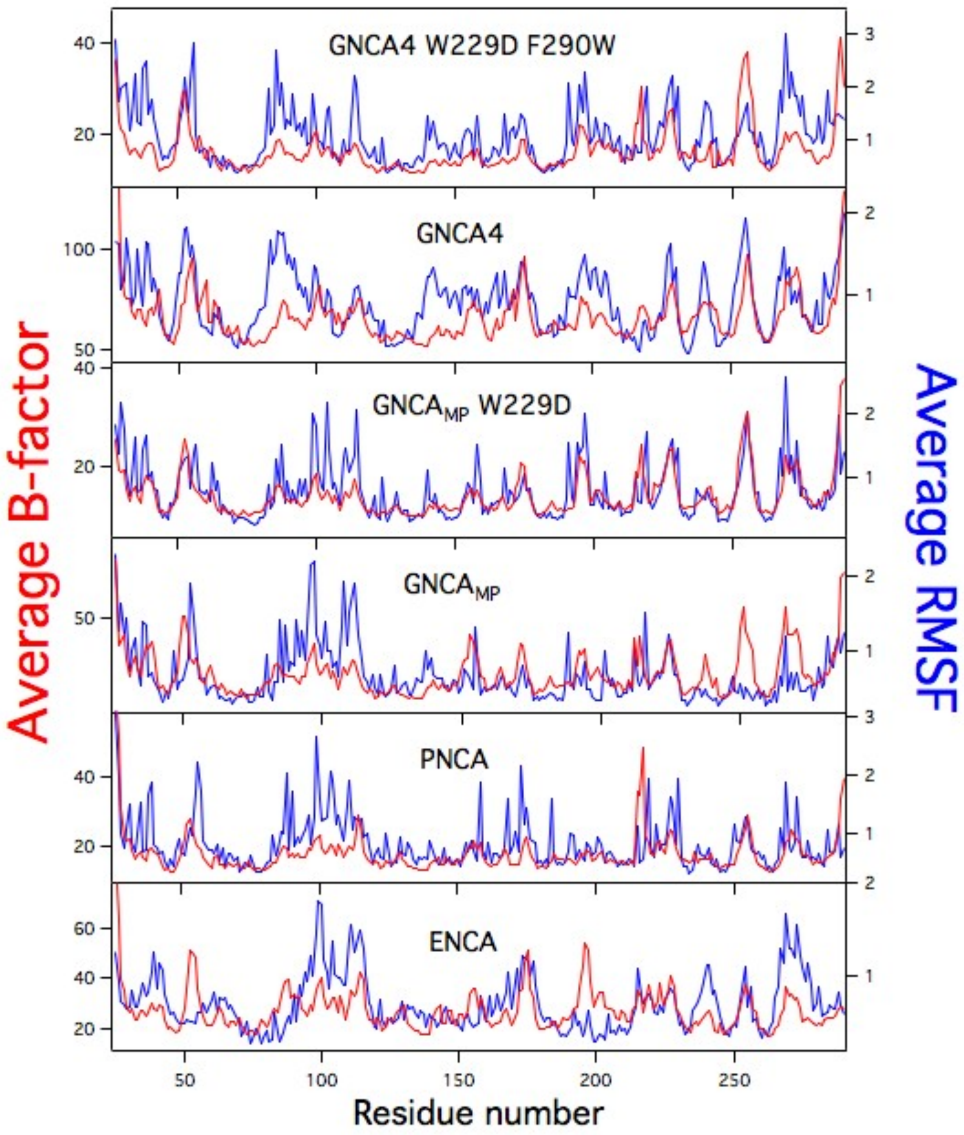

Supplementary Figure 28. Structural Validation of Ancestral β-Lactamase Simulations Comparison between root mean square fluctuations and crystallographic B-factors for several resurrected Precambrian β-lactamases studied in this work. The average residue values for the RMSF and B-factors are plotted *vs*. the residue number. The overall congruence observed (regions of high RMSF often, although not always, match regions of high B-factor) appears comparable to that reported by Orozco and co-workers in their consensus analysis of protein dynamics (see Figure 3 in ref. [4]).

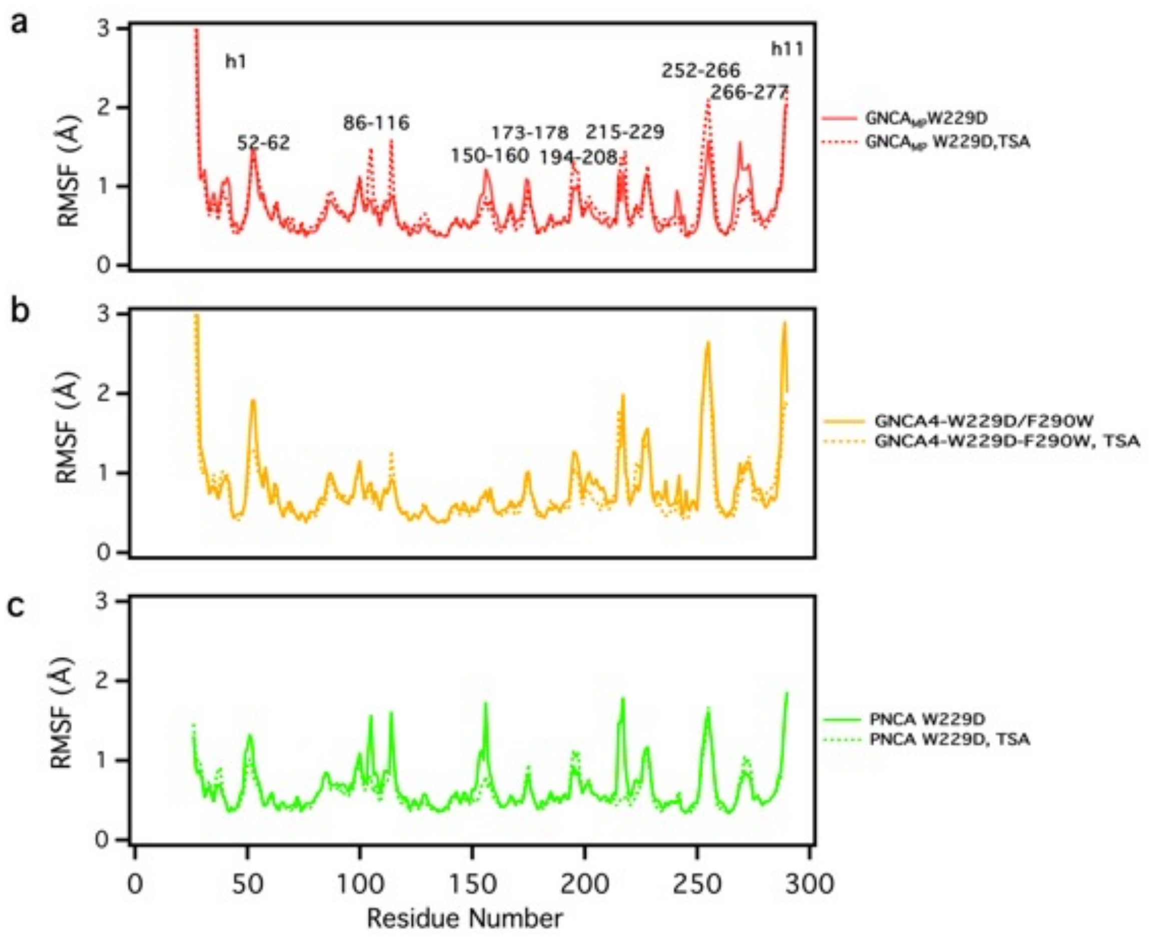

Supplementary Figure 29. Additional C_α Root Mean Square Deviations
Root mean square fluctuations (RMSF), in Å, of all C_α atoms during our simulations of (A) the $GNCA_{MP}$, (B) GNCA4 and (C) PNCA β-lactamases both in the absence (solid lines) and presence (dashed lines) of the transition state analogue (TSA) 5(6)-nitrobenzotriazole. All values shown here are averages over the last 60 ns of three independent trajectories (180 ns total simulation time), obtained as described in the Methods section of the main text.

SUPPLEMENTARY TABLES

Supplementary Table 1. Sequence Identity During the Evolution of β-Lactamases[a]

	ENCA	GPBCA	GNCA	AFCA	FCA	PNCA
**ENCA**	100					
**GPBCA**	69	100				
**GNCA**	62	88	100			
**AFCA**	54	72	79	100		
**FCA**	40	51	54	64	100	
**PNCA**	60	84	94	82	56	100

[a] Shown here is the percentage of sequence identity between the most probabilistic sequences at Precambrian nodes in the evolution of β-lactamases. See Figure 1 in the main text for node definitions.

Supplementary Table 2. Amino Acid Differences During the Evolution of β-Lactamases[a]

	$GNCA_{MP}$	GNCA 1	GNCA 2	GNCA 3	GNCA 4	GNCA 5	GNCA 6	GNCA 7
**$GNCA_{MP}$**	0							
**GNCA1**	9	0						
**GNCA2**	20	22	0					
**GNCA3**	9	8	20	0				
**GNCA4**	15	12	26	11	0			
**GNCA5**	9	14	15	12	19	0		
**GNCA6**	8	9	16	9	17	9	0	
**GNCA7**	11	11	19	11	16	12	11	0

[a] Shown here is the number of amino acid differences between different reconstructed sequences at the GNCA node in the evolution of β-lactamases. See Figure 1 for node definitions. Additional information on these proteins can be found in Supplementary Table 3.

Supplementary Table 3. Mutational Differences Between GNCA Node β-Lactamases

Mutation	GNCA1	GNCA2	GNCA3	GNCA4	GNCA5	GNCA6	GNCA7	Distance Cα-Cα (Å)	Distance between closest side-chain atoms (Å)
Q28A	X		X	X				22.5	15.6
Q28K		X					X	22.5	15.6
L29A		X						19.6	10.7
S30A		X						22.4	16.7
Q32R	X							22.0	13.8
E35A		X				X	X	25.4	20.1
A47S		X						18.4	14.3
I57F	X			X			X	19.9	12.1
A58G			X	X				21.4	16.6
R90N				X				32.2	32.6
G98S		X			X	X		40.4	40.4
E100A		X	X		X			41.2	41.2
L129M			X		X		X	21.1	21.1
L129Y				X				21.1	21.1
A141S	X	X			X	X		34.4	34.1
I155L		X	X			X	X	28.7	25.8
E158N				X				31.4	29.1
E171T		X		X				32.8	31.7
A198V	X		X	X		X		24.9	24.6
V208T		X						14.1	12.4
V212I				X	X		X	10.7	9.9
K215T		X						16.1	16.3
G240E				X				30.8	30.8
H241Y		X						31.5	29.7
G255N		X		X				9.7	9.7
I259L		X						10.8	5.2
V260L	X	X	X	X			X	12.9	10.3
T262A	X		X	X		X	X	16.4	13.6
S268A		X			X			28.4	24.4
V270A		X			X		X	28.3	24.0
G283A	X	X	X	X	X	X		11.3	11.3
V287A							X	10.1	3.8
E288A				X				13.6	7.1

[a] Shown here are the mutational differences between the alternative representations of β-lactamases corresponding to the GNCA node (GNCA**1** to GNCA**7**) and the most probabilistic sequence at that node ($GNCA_{MP}$ β-lactamase). The sequence of the $GNCA_{MP}$ β-lactamase is given in Risso et al. (2013)[5] and has 122 mutational differences with that of the modern TEM-1 β-lactamase. The alternative representations of the sequence at the GNCA node were derived from Monte Carlo sampling of the posterior probability distribution, as described by Risso et al. (2013)[5]. The distances between the each mutated residue and D229 in the structure of $GNCA_{MP}$-W229D lactamase are given.

Supplementary Table 4. Sequence Identity Between Modern β-Lactamases of Interest[a]

	TEM-1	BL	1HZO	1G68	1E25	1BZA	2CC1	1BUE	1BSG	3BLM
**TEM-1**	100									
**BL**	35	100								
**1HZO**	35	41	100							
**1G68**	42	28	34	100						
**1E25**	21	22	20	22	100					
**1BZA**	35	39	69	36	23	100				
**2CC1**	38	38	40	31	23	41	100			
**1BUE**	33	40	45	33	21	47	37	100		
**1BSG**	37	40	40	30	21	41	41	40	100	
**3BLM**	31	40	33	34	19	34	31	33	30	100

[a] Percentage of sequence identity between the sequences of the 10 modern β-lactamases studied in this work. TEM-1 and BL stand for TEM-1 and *B. licheniformis* β-lactamases. All other modern lactamases (see Figure 1 of the main text) are identified by their PDB codes.

Supplementary Table 5. Percentage Sequence Identity Between Key β-Lactamases[a]

	TEM-1	BL	1HZO	1G68	1E25	1BZA	2CC1	1BUE	1BSG	3BLM
**ENCA**	79	36	39	45	23	40	42	38	41	29
**GPBCA**	58	43	50	47	27	52	47	45	52	35
**$GNCA_{MP}$**	54	45	52	42	29	55	49	46	54	35
**GNCA1**	53	45	51	40	30	53	48	46	54	35
**GNCA2**	52	45	53	41	30	56	49	48	55	35
**GNCA3**	54	44	52	41	30	55	49	45	53	35
**GNCA4**	53	44	53	40	30	54	48	47	52	35
**GNCA5**	54	45	52	42	29	55	49	46	55	36
**GNCA6**	54	45	52	41	30	55	48	45	54	36
**GNCA7**	54	45	51	42	29	55	48	46	54	35
**FCA**	40	67	48	35	22	50	38	47	45	44
**AFCA**	47	52	52	38	27	56	52	47	59	38
**PNCA**	53	47	52	42	30	56	49	46	54	36

[a] Percentage of sequence identity between sequences of the 10 modern β-lactamases and the sequences of the 13 ancestral β-lactamases studied in this work. See Figure 1 fin the main text or the definition of the ancestral nodes. TEM-1 and BL stand for TEM-1 and B. licheniformis β-lactamases. All other modern lactamases are identified by their PDB codes.

Supplementary Table 6. Kemp Elimination by Ancestral and Modern β-Lactamases[a]

Variant	k_{cat}/K_M (M^{-1} s^{-1})	k_{cat} (s^{-1})	K_M (mM)
$GNCA_{MP}$-W229D	45±6	>0.5	ND
$GNCA_{MP}$-W229D (no His-tag)	38±2	>0.5	ND
GNCA1-W229D	156±2	>0.2	ND
GNCA2-W229D	45±5	>0.5	ND
GNCA3-W229D	320±9	>1.7	ND
GNCA4-W229D	250±7	>8	ND
GNCA5-W229D	180±7	>3	ND
GNCA6-W229D	128±3	>0.5	ND
GNCA7-W229D	14.9±0.3	>0.4	ND
$GNCA_{MP}$-D228A/W229D	34±4	ND	ND
GPBCA-W229D	9.7±0.6	>0.2	ND
PNCA-W229D	4.1±0.5	>0.02	ND
FCA-W229D	175±22	>0.2	ND
AFCA-W229D	2.30±0.03	>0.02	ND
$GNCA_{MP}$-W229D/F290W	201±40	0.7±0.06	3.4±0.4
GNCA1-W229D/F290W	289±21	0.6±0.12	2.1±0.5
GNCA2-W229D/F290W	106±15	0.1±0.03	1.1±0.4
GNCA3-W229D/F290W	986±49	1.7±0.29	1.7±0.3
GNCA4-W229D/F290W	3047±283	3.7±0.57	1.2±0.3
GNCA4-W229D/F290W (high pH)	5497±600	12±2	1.8±0.6
GNCA4-W229D/F290W (no His-tag)	1705±139	2.6±0.44	1.5±0.4
GNCA5-W229D/F290W	790±82	1.3±0.28	1.6±0.1
GNCA6-W229D/F290W	947±57	3.5±1.00	3.7±1.2
GNCA7-W229D/F290W	120±11	0.2±0.04	1.8±0.5

[a] Shown here are the catalytic parameters for Kemp eliminase activities of ancestral and modern β-lactamase variants. All values provided are derived from the analysis of profiles of rate *vs.* substrate concentration determined at pH 7 (see Figure 7 in the main text as well as Supplementary Figures 24 and 25). For linear Michaelis plots, lower limit estimates of k_{cat} were calculated as explained in Supplementary Figure 5.

Supplementary Table 7. Antibiotic Hydrolysis by the GNCA β-lactamases[a]

	$GNCA_{MP}$-W229D	GNCA4-W229D/F290W
BZ		
k_{cat} (s^{-1})	5.6±1	4.7±0.9
k_{cat}/K_M (s^{-1} μM^{-1})	0.6±0.3	0.15±0.1
CTX		
k_{cat} (s^{-1})	82.0±7.3	24.4±5.3
k_{cat}/K_M (s^{-1} μM^{-1})	0.80±0.32	1.1±0.4

[a] Shown here are the Michaelis-Menten parameters for the hydrolysis of several antibiotics catalyzed by some of the β-lactamases with Kemp eliminase activity studied in this work. BZ denotes benzylpenicillin and CTX denotes cefotaxime.

Supplementary Table 8. X-Ray Data Collection and Refinement Statistics

**Protein**<br>**Mutant**<br>**TS analog**	GNCA4	GNCA4<br>W229D/F290W	GNCA4<br>W229D/F290W<br>analog bound	$GNCA_{MP}$	$GNCA_{MP}$<br>W229D	$GNCA_{MP}$<br>W229D<br>analog bound
**PDB ID.**	5FQQ	5FQI	5FQK	5FQM	4UHU	5FQJ
**Data Collection**						
Beam-line	ID29 (ESRF)	ID29 (ESRF)	Xaloc (ALBA)	ID29 (ESRF)	ID23-1 (ESRF)	ID30A (ESRF)
Resolution range	42.73 - 2.12 (2.196 - 2.12)	37.36-1.4 (1.45-1.4)	42.14 - 1.767 (1.83 - 1.767)	21.04 - 1.5 (1.554 - 1.5)	26.56 - 1.305 (1.352 - 1.305)	41.3 - 2.274 (2.355 - 2.274)
Space group	P 61	P 61	P 61	I 41	P 21 21 2	P 21 21 2
Cell dimensions a, b, c (Å)	49.34, 49.34, 199.11	46.96, 46.96, 189.13	49.83, 49.83, 196.094	94.23, 94.23, 93.58	70.14, 50.57, 69.72	51.17, 69.79, 69.96
Unique reflections	15497 (1556)	46046 (4575)	26853 (2680)	59508 (5318)	60143 (5788)	11969 (1116)
Multiplicity	5.5 (5.7)	3.3 (3.2)	20.7 (19.5)	1.9 (1.9)	1.9 (1.9)	6.5 (6.6)
Completeness (%)	1.00 (1.00)	1.00 (0.99)	1.00 (1.00)	0.91 (0.82)	0.99 (0.96)	0.99 (0.95)
Mean I/sigma(I)	15.91 (1.61)	10.49 (1.75)	28.08 (2.48)	3.58 (1.27)	16.93 (2.51)	9.66 (1.85)
Wilson B-factor	58.80	16.33	36.49	16.05	12.65	34.11
R-merge	0.050 (0.95)	0.054 (0.54)	0.07 (1.33)	0.12 (0.54)	0.027 (0.34)	0.16 (1.11)
CC1/2	0.999 (0.679)	0.995 (0.709)	1 (0.947)	0.964 (0.28)	0.999 (0.742)	0.995 (0.76)
**REFINEMENT**						
R-work	0.19 (0.31)	0.15 (0.26)	0.18 (0.27)	0.19 (0.27)	0.16 (0.29)	0.20 (0.28)
R-free	0.23 (0.31)	0.17 (0.27)	0.20 (0.30)	0.20 (0.31)	0.18(0.29)	0.23 (0.28)
Non-hydrogen atoms	2074	2422	2221	2503	2418	2192
Macromolecules	2031	2138	2126	2121	2136	2111
Ligands	15	96	12	71	17	12
Water	28	188	83	311	265	69
Protein residues	262	263	263	262	264	265
RMS(bonds)	0.008	0.024	0.003	0.010	0.006	0.006
RMS(angles)	1.55	0.92	0.73	1.39	1.13	0.86
Ramachandran Favored (%)	96	99	98	98	99	96
Outliers (%)	0.38	0	0.37	0	0	0
Average B-factors ($Å^2$)	74.65	22.79	55.64	22.11	18.21	40.43
Macromolecules	74.74	20.83	55.51	18.98	16.27	40.30
Ligands	91.22	44.69	100.91	40.61	34.70	63.38
Solvent	59.55	33.86	52.48	39.30	32.84	40.60

Supplementary Table 9. NMR Relaxation Parameters for TEM-1 and $GNCA_{MP}$

	TEM-1	$GNCA_{MP}$
R_1 (s^{-1})	1.04 ± 0.03	1.13 ± 0.11
R_2 (s^{-1})	17.10 ± 0.58	16.2 ± 1.1
R_2/R_1	16.52 ±1.11	14.8 ± 1.5
$\{^1H\}$-^{15}N NOE	0.79 ± 0.04	0.79 ± 0.06

[a] Comparison of the NMR relaxation parameters for the TEM-1[6] and $GNCA_{MP}$ (this work) β-lactamases. See Supplementary Methods for details

Supplementary Table 10. Dynamic Values from NMR for TEM-1 and $GNCA_{MP}$

	TEM-1	$GNCA_{MP}$
S^2 average	0.90 ± 0.02	0.91 ± 0.06
τ_m (ns)	12.41 ± 0.01	11.68 ± 0.02
D_{par}/D_{per}	1.23 ±0.01	1.27 ± 0.01

[a] Comparison of the dynamic values for the TEM-1[6] and $GNCA_{MP}$ (this work) lactamases as obtained from the NMR relaxation data. See Supplementary Methods for details.

Supplementary Table 11. Number of Residues Described by Different Dynamic Models

	TEM-1 (residues)	$GNCA_{MP}$ (residues)
Model 1 S^2	147/222	125/177
Model 2 S^2, τ_e (sub-nanosec) fast scale	42	16
Model 3 S^2, R_{ex} (micro-milisec) slow scale	6	25
Model 4 S^2, τ_e, R_{ex}	6	1
Model 5 two time-scales contribution	4	5
None	17	4

[a] Comparison of the number of residues that can be described by the different dynamic models in the model-free formalism, for the TEM-1 and $GNCA_{MP}$ lactamases. See Supplementary Methods for details.

Supplementary Table 12. Non-Standard Force Field Parameters Used in This Work

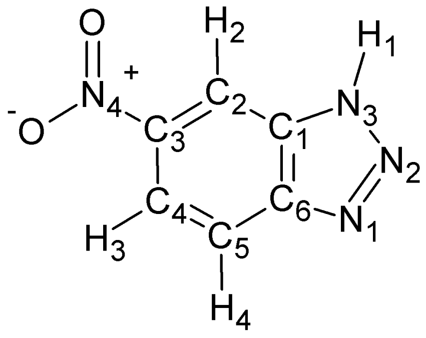

Atom Name	Atom Type	Partial Charge
N1	nc	-0.3735
N2	nd	-0.1606
C1	ca	0.1118
N3	na	-0.0828
C2	ca	-0.3598
C3	ca	-0.0173
C4	ca	-0.1342
C5	ca	-0.4034
C6	ca	0.5452
O1	o	-0.4821
O2	o	-0.4821
N4	no	0.8652
H1	hn	0.3047
H2	ha	0.2488
H3	ha	0.1973
H4	ha	0.2229

Dihedral Angle	Barrier height V_n (kcal•mol^{-1})	Phase γ (°)	Periodicity (n)	Type
ca-ca-no-o	6.0	180.0	2.0	Proper
ca-ca-ca-na	1.1	180.0	2.0	Improper
ca-hn-na-nd	1.1	180.0	2.0	Improper
ca-ca-ca-ha	1.1	180.0	2.0	Improper
ca-ca-ca-no	1.1	180.0	2.0	Improper
ca-ca-ca-nc	1.1	180.0	2.0	Improper
ca-o-no-o	6.0	180.0	2.0	Improper

[a] Non-standard force field parameters for the transition-state analog 5(6)-nitrobenzotriazole (TSA), obtained as described in the Materials and Methods section of the main text. The torsion potential energy function used is: $E_{dihe} = 0.5*V_n(1+\cos(n*\Phi-\gamma)$.

SUPPLEMENTARY NOTE

Can static structural features of the modern lactamases explain their inefficiency as backgrounds for the generation of Kemp eliminase activity?

Experiments performed to explore whether some static structural features of the modern TEM-1 background can explain its inefficiency as a scaffold for the *de novo* generation of Kemp eliminase activity on the basis of a single W229D mutation.

The rationale behind the experiments performed is made apparent by visual inspection of the superposition between the structures of TEM-1 lactamase and our best Kemp eliminase (GNCA4-W229D/F290W), shown in panel A of Supplementary Figure 20. The loop formed by residues 252-258 clearly has a different conformation in the two scaffolds. That is in the modern background, the loop shifts away from position 229 and, therefore, it is unlikely to cause steric interference. Still, we deemed it convenient to mutationally probe the loop position that is closer to W229: position 254, at which the ancestral residue is proline and the modern residue is aspartate. However, the D254P mutation did not confer significant *de novo* Kemp eliminase activity to the TEM1-W229D/M182T lactamase.

More remarkably, we noted that, although the residue at position 290 is tryptophan in both the modern TEM-1 lactamase and the ancestral/engineered GNCA2-W229D/F290W, its spatial orientation is quite different. This is likely due to a cation-π interaction with R259 in the modern protein. Note that such a cation-π interaction is not possible in the ancestral background because the residue at the corresponding position 259 in the GNCA lactamase is an isoleucine. We speculated, therefore, that the orientation of the tryptophan at position 290 could hamper substrate and transition state binding to the cavity created by the W229D mutation in the modern background, thus precluding the generation of Kemp eliminase activity. To mutationally probe this possibility we carried out the following studies: 1) We performed an R259I mutation in the TEM1-W229D/M182T background. This mutation should eliminate the R-W cation-π interaction in the modern background, thus allowing the re-orientation of the W290 residue and perhaps leading to the generation of Kemp eliminase activity. However, we found that the R259I mutation did not generate any significant de novo activity in the TEM1-W229D/M182T lactamase. 2) We mutated W290 in TEM1-W229D/M182T to the ancestral F residue in that position, but observed no significant Kemp eliminase activity. 3) Finally, we mutated W290 in TEM1-W229D/M182T to alanine and glycine in order to remove any steric hindrance to catalysis linked to residue W290 in the modern scaffold. However, despite being stable (with a denaturation temperature of about 55 ºC from DSC), both the W290G and W290A variants of TEM1-W229D/M182T lactamase were as inactive for Kemp elimination as the parent TEM1-W229D/M182T background.

Experiments performed to explore whether some static structural features of the modern *Bacillus licheniformis* β-lactamase can explain its inefficiency as a scaffold for the *de novo* generation of Kemp eliminase activity on the basis of a single W229D mutation.

The experiments performed targeted the role of the residue at position 291. Structural superposition (panel B of Supplementary Fig. 20) indicates that residue 291 in the *Bacillus licheniformis* β-lactamase corresponds to residue 290 in the TEM-1 and GNCA β-lactamases. N291 was mutated to A to test the possibility that steric interference prevents catalysis. Mutations to F and W were performed because these are the residues present at the

corresponding position in active variants of GNCA lactamases. Mutation to L was performed because this residue is present at the corresponding position in the active variant of the FCA lactamase. Although some small activity increase was observed to occur upon these mutations, all the activity levels were barely indistinguishable from the blanks. A small steric interference between the transition state and the residue at position 287 (a methionine) is also suggested by the superposition in panel B of Supplementary Fig. 20. However, mutation to alanine (to eliminate the potential interference) does not lead to a significant level of Kemp elimination activity. On the other hand, we did find that a back-to-the-ancestor mutation (M287V, where valine is the residue present at position 287 in FCA lactamase) does lead to a significant activity level.

SUPPLEMENTARY METHODS

NMR spectroscopy of the GNCA$_{MP}$ lactamase

Materials and Methods

NMR sequential assignment.

All NMR experiments were performed at 31.5 ºC on a Bruker AV 800 spectrometer equipped with a cryoprobe on a 0.6 mM uniformly ^{13}C,^{15}N-labeled sample. Sequence-specific assignments were made using standard procedures with the following experiments: 2D ^{1}H-^{15}N HSQC and 3D HNCO, HN(CA)CO, HN(CO)CA, HNCAi, CBCA(CO)NH and HNCACB[7]. Data obtained with these experiments were complemented with those of specific amino acid type discrimination[8,9]. NMRPipe[10] and Sparky[11] were used to process raw NMR data and for interactive spectrum analysis, respectively. Chemical shifts were referenced to the water signal as an internal reference for ^{1}H using pH and temperature corrections[12,13]. ^{15}N and ^{13}C chemical shifts were referenced indirectly[14].

^{15}N Relaxation Measurements and Analysis.

^{15}N relaxation parameters T_1, $T_1\rho$, T_2 and $\{^{1}H\}$-^{15}N NOE[15] were acquired on a Bruker AV 600 spectrometer equipped with a cryoprobe, at 31.5 ºC on a 0.6 mM, buffered pH 6.7, uniformly ^{15}N-labeled sample. Twelve delays (20, 60, 100, 160, 240, 460, 640, 860, 1260, 1600, 2200 and 2750 ms) were used for T_1 measurements, ten delays (8, 16, 36, 56, 76, 100, 128, 156, 180 and 200 ms) were used for $T_1\rho$ determinations, and a different set of twelve delays (0, 16, 31, 47, 63, 80, 96, 111, 127, 142, 174 and 190 ms) was used to measure the T_2 values. The recycle delay was 3.0 s in all experiments. $\{^{1}H\}$-^{15}N NOE experiments were carried out with an overall recycling delay of 10 s to ensure the maximal development of NOEs before acquisition and to allow solvent relaxation, thus avoiding transfer of saturation to the most exposed amide protons of the protein between scans[16]. Relaxation times were calculated *via* least-squares fitting of peak intensities to a two-parameter exponential function, using the rate analysis routine of the java version of NMRView[17]. Heteronuclear NOEs were calculated from the ratio of cross-peak intensities in spectra collected with and without amide proton saturation during the recycle delay. Uncertainties in peak heights were determined from the standard deviation of the distribution of intensities in the region of the HSQC spectra where no signal was present and only noise was observed.

Internal Dynamics.

The principal components of the $GNCA_{MP}$ inertia tensor were calculated with the Pdbinertia program[18] using the X-ray structure of the $GNCA_{MP}$ lactamase[5]. We estimated the overall correlation time from the ratio of the mean T_1 and T_2 values. These mean values of T_1, $T_1\rho$ and T_2 were calculated from a subset of residues with little internal motion and no significant exchange broadening. This subset excluded residues with NOE values lower than 0.65 and also residues with T_2 values lower than the average minus one standard deviation, unless their corresponding T_1 values were larger than the average plus one standard deviation[19]. The diffusion tensor, which describes rotational diffusion anisotropy, was determined by two approaches[20,21], with the r2r1_diffusion and the quadric_diffusion programs[22]. The calculations were unsuccessful after using the errors in T_1 and T_2 estimated by Monte Carlo simulations; these were unrealistically low. Therefore, the errors were scaled up by the minimum factor allowing an interpretation of the data in terms of a rotational diffusion tensor. This procedure resulted in 5% average errors. The ^{15}N relaxation was analyzed assuming dipolar coupling with the directly attached proton (with a bond length of 1.02 Å), and a contribution from the ^{15}N chemical shift anisotropy evaluated as -172 ppm. Relaxation data were fitted to the Lipari and Szabo model using FAST-Modelfree[17], with interfaces with MODELFREE version 4.2[23]. Five models of internal motion were evaluated for each amide ^1H-^{15}N pair, each one described by the following parameters: S^2, S^2 and τ_e, S^2 and R_{ex}, S^2, τ_e, and R_{ex}, and S_f^2, S_s^2, and τ_e; where S^2 is the generalized order parameter of the internal mition, τ_e is the effective internal correlation time, R_{ex} is the exchange contribution to transverse relaxation, and S_f^2 and S_s^2 are related to the amplitude of the fast and slow internal motions. Model v takes into account a situation with two distinctive internal motions (with at least 2 or 3 orders of magnitude between their time constants) both faster than τ_m (overall correlation time). The order parameters S_f^2 (fast ps) and S_s^2 (slow ns) reflect the amplitude of the two internal motions, being τ_e the time constant for the slower one.

In order to get a good comparison with a related protein, the same approach was applied to the NMR relaxation parameters published for TEM-1[6].

NMR Assignments of $GNCA_{MP}$.

^1H and ^{15}N assignments were obtained by the combination of triple resonance spectra. Supplementary Figure 21 shows a ^1H-^{15}N-HSQC spectrum of $GNCA_{MP}$ and the ^1H and ^{15}N assignments are reported on Supplementary Data. The only unassigned residues in $GNCA_{MP}$ were Ala26, Ala27, Ser70 and Thr237.

Backbone Dynamics from ^{15}N Relaxation of $GNCA_{MP}$.

We have measured a large set of individual ^{15}N relaxation parameters for the $GNCA_{MP}$ lactamase (Supplementary Figure 22). The heteronuclear $\{^1H\}$-^{15}N NOE and the longitudinal (T_1) and transversal (T_2) relaxation times were measured for the 71% of the total 262 residues. The exceptions are the N-terminal residue, the twelve prolines, and some others due to severe signal overlapping in the crowded NMR spectra.

The average values of the ^{15}N relaxation parameters are summarized in Supplementary Table 9. There were several residues that deviated from the average. These are mostly located at the C-terminus, and at some loops. In these cases, low NOE values (Supplementary Fig. 21) indicate flexibility in the fast time scale (picoseconds to nanoseconds). The average value of the order parameter (S^2) is 0.91± 0.05 showing that, globally, the GNCA lactamase has a high degree of order on the pico-to-nanosecond time scale. In general, residues in loop regions have lower values.

Similar results were found in the related protein TEM-1[6]. The calculated global rotational diffusion correlation time (τ_m) for the $GNCA_{MP}$ lactamase was 11.68 ± 0.02 ns. This value is in good agreement with the value obtained by hydrodynamic calculations (12.27 ns) (see Supplementary Table 10).

The principal components of the inertia tensor, calculated for the X-ray structure[5], have relative values of 1.00, 0.90, and 0.60. These values indicate that the shape deviates from that of a sphere and approaches a prolate ellipsoid. In agreement with these findings, the diffusion tensor that better explained the NMR relaxation data was anisotropic, with different values for the two components (parallel and orthogonal) of the tensor giving a value of $D\|/D\perp$ of 1.27 ± 0.15 (Supplementary Table 10).

On the bases of all these results, the relaxation data for the amide ^{1}H-^{15}N pair of each residue were analyzed using the model-free formalism to calculate the corresponding dynamical parameters. Most data (125+16 spins) could be satisfactorily described by one of the two simpler dynamical models (see Methods in the main text) (Supplementary Table 11), which describe the internal dynamics of the ^{1}H-^{15}N pair in terms of a generalized order parameter S^2 and an effective internal correlation time τ_e of fast motions, which is always faster than the global correlation time. In a significant number of cases (twenty-six 25+1 residues), it was necessary to include a contribution of the slow motions to the transverse relaxation time, on the microsecond to millisecond time scale. In these cases, the internal dynamics is characterized by the contribution of conformational exchange, R_{ex}. Even though data of exchange contribution was estimated from measurements at a single field, it is important to emphasize that the R_2/R_1 ratio is very homogeneous (Supplementary Figure 22), and deviations from the expected correlation were not detected. Moreover, the rotational diffusion tensor was analysed using the full relaxation dataset in combination with the crystallographic structure of the mutant to rule out that an increased R_2 value would be induced by the molecular anisotropy.

In a few cases (five residues), the inclusion of the amplitude of two internal motions (S_f^2 and S_s^2) was also necessary to obtain a good fitting. Finally, only four residues were not fitted to any model.

SUPPLEMENTARY REFERENCES

1. Bevington, P. R. 1969. *"Data reduction and Error Analysis for the Physical Sciences"*; McGraw-Hill; New York.

2. Kikuchi, K., Thorn, S.N. & Hilvert, D. Albumin-catalyzed proton transfer. *J. Am. Chem. Soc.* **118,** 8184-8185 (1996).

3. Hollfelder, F., Kirby, A. J., Tawfik, D. S., Kikuchi, K. & Hilvert, D. Characterization of proton transfer catalysis by serum albumins. *J. Am. Chem. Soc.* **122,** 1022-1029 (2000).

4. Rueda, M. *et al.* A consensus view of protein dynamics. *Proc. Natl. Acad. Sci. USA* **104,** 796-801 (2007).

5. Risso, V. A., Gavira, J. A., Mejia-Carmona, D. F., Gaucher, E. A. & Sanchez-Ruiz, J. M. Hyperstability and substrate promiscuity in laboratory resurrections of Precambrian β-lactamases. *J. Am. Chem. Soc.* **135**, 2899-2902 (2013).

6. Savard, P. Y. & Gagné, S. M. Backbone dynamics of TEM-1 determined by NMR: evidence for a highly ordered protein. *Biochemistry* **45**, 11414-11424 (2006).

7. Sattler, M., Schleucher, J. & Griesinger, C. Heteronuclear multidimensional NMR experiments for the structure determination of proteins in solution employing pulsed field gradients. *Prog. Nucl. Magn. Reson. Spetrosc.* **34**, 93-158 (1999).

8. Pantoja-Uceda, D. & Santoro, J. Amino acid type identification in NMR spectra of proteins via beta- and gamma-carbon edited experiments. *J. Magn. Reson.* **195**, 187-195 (2008).

9. Pantoja-Uceda, D. & Santoro, J. A suite of amino acid residue type classification pulse sequences for 13C-detected NMR of proteins. *J. Magn. Reson.* **234**, 190-196 (2013).

10. Delaglio, F. *et al.* NMRPipe: a multidimensional spectral processing system based on UNIX pipes. *J. Biomol. NMR* **6**, 277-293 (1995).

11. Goddard, T. D.; Kneller, D. G. SPARKY 3; University of California; San Francisco.

12. Hartel, A. J., Lankhorst, P. P. & Altona, C. Thermodynamics of stacking and of self-association of the dinucleoside monophosphate m2(6)A-U from proton NMR chemical shifts: differential concentration temperature profile method. *Eur. J. Biochem.* **129**, 343-357 (1982).

13. Orbons, L. P., van der Marel, G. A., van Boom, J. H. & Altona, C. An NMR study of polymorphous behaviour of the mismatched DNA octamer d(m5C-G-m5C-G-A-G-m5C-G) in solution. The B-duplex and hairpin forms. *Eur. J. Biochem.* **170**, 225-239 (1987).

14. Wishart, D. S. *et al.* 1H, 13C and 15N chemical shift referencing in biomolecular NMR. *J. Biomol. NMR* **6**, 135-140 (1995).

15. Farrow, N. A. *et al.* Backbone Dynamics of a Free and a Phosphopeptide-Complexed Src Homology 2 Domain Studied by 15N NMR Relaxation. *Biochemistry* **33**, 5984-6003 (1994).

16. Renner, C., Schleicher, M., Moroder, L. & Holak, T. A. Practical aspects of the 2D 15N-[1h]-NOE experiment. *J. Biomol. NMR* **23,** 23-33 (2002).

17. Johnson, B. A. & Blevins, R. A. NMRView: A computer program for the visualization and analysis of NMR data. *J. Biomol. NMR* **4**, 603-614 (1994).

18. Palmer, A.G. III; Columbia University; New York; NY.

19. Pawley, N. H., Wang, C., Koide, S. & Nicholson, L. K. An improved method for distinguishing between anisotropic tumbling and chemical exchange in analysis of ^{15}N relaxation parameters. *J. Biomol. NMR* **20**, 149-165 (2001).

20. Bruschweiler, R., Liao, X. & Wright, P. E. Long-range motional restrictions in a multidomain zinc-finger protein from anisotropic tumbling. *Science* **268**, 886-889 (1995).

21. Tjandra, N., Feller, S. E., Pastor, R. W. & Bax, A. Rotational diffusion anisotropy of human ubiquitin from 15N NMR relaxation *J. Am. Chem. Soc.* **117**, 12562-12566 (1995).

22. Cole, R. & Loria, J. P. FAST-Modelfree: a program for rapid automated analysis of solution NMR spin-relaxation data. *J. Biomol. NMR* **26**, 203-213 (2003).

23. Palmer, A. G., Rance, M. & Wright, P. E. Intramolecular motions of a zinc finger DNA-binding domain from xfin characterized by proton-detected natural abundance ^{13}C heteronuclear NMR spectroscopy. *J. Am. Chem. Soc.* **113**, 4371-4380 (1991).
