# Supplementary material for: De novo active sites for resurrected Precambrian enzymes
Source: Nat Commun. 2017 Jul 18;8:16113. doi: 10.1038/ncomms16113 (PMC5520109; doi:10.1038/ncomms16113)
Supplement: Supplementary Data 1 [file ncomms16113-s2.docx]

**Supplementary Data.** Backbone ^1^H and ^15^N assignments (ppm) of GNCA_MP_ lactamase.

| **Residue** | **GNCA_MP_** | | **Residue** | **GNCA_MP_** | |
| --- | --- | --- | --- | --- | --- |
|  | **^1^H** | **^15^N** |  | **^1^H** | **^15^N** |
| A26 |  |  | A78 | 117.3 | 7.16 |
| A27 |  |  | A79 | 119.4 | 7.82 |
| Q28 | 120.2 | 8.57 | V80 | 118.9 | 7.7 |
| L29 | 123.7 | 8.57 | L81 | 120.3 | 8.26 |
| S30 | 112.7 | 8.53 | A82 | 122 | 8.34 |
| E31 | 121.4 | 7.46 | R83 | 116.8 | 7.23 |
| Q32 | 119.4 | 7.97 | V84 | 124.1 | 8.1 |
| L33 | 120.5 | 8.34 | D85 | 123.7 | 8.97 |
| A34 | 121.6 | 7.37 | Q86 | 115.2 | 7.44 |
| E35 | 118.7 | 7.45 | G87 | 108.7 | 8.29 |
| L36 | 122.1 | 7.69 | K88 | 117.4 | 8.13 |
| E37 | 119.3 | 8.45 | E89 | 118.8 | 7.37 |
| K38 | 120.9 | 7.86 | R90 | 125 | 10.1 |
| R39 | 119 | 7.81 | L91 | 121.1 | 8.89 |
| S40 | 113.8 | 8.09 | D92 | 111.6 | 8.04 |
| G41 | 110.3 | 7.67 | R93 | 121.8 | 7.12 |
| G42 | 107.5 | 7.93 | R94 | 127.4 | 8.52 |
| R43 | 124.5 | 9.06 | I95 | 131.3 | 9.14 |
| L44 | 130.1 | 10.2 | T96 | 118 | 8.03 |
| G45 | 112.6 | 9.44 | Y97 | 119 | 7.78 |
| V46 | 123.1 | 9.18 | G98 | 107.1 | 9.36 |
| A47 | 126.1 | 8.77 | K99 | 120 | 8.73 |
| V48 | 119.7 | 8.67 | E100 | 117.6 | 9.51 |
| L49 | 127.7 | 9.17 | D101 | 117.4 | 7.67 |
| D50 | 125.1 | 8.8 | L102 | 116.4 | 7.06 |
| T51 | 116.2 | 7.82 | V103 | 117.2 | 7.75 |
| A52 | 123.6 | 8.43 | D104 | 122.6 | 8.27 |
| T53 | 102.8 | 7.02 | Y105 | 121 | 8.49 |
| G54 | 111.3 | 8.23 | S106 | 120.5 | 8.34 |
| R55 | 121.6 | 7.76 | P107 |  |  |
| R56 | 123.7 | 8.41 | V108 | 117.9 | 8.51 |
| I57 | 123.7 | 8.77 | T109 | 113.2 | 9.44 |
| A58 | 126.4 | 8.57 | E110 | 120.3 | 8.21 |
| Y60 | 124.2 | 9.34 | K111 | 116.2 | 7.29 |
| R61 | 123.1 | 8.61 | H112 | 114.5 | 7.12 |
| G62 | 99.8 | 8.17 | V113 | 118.9 | 7.19 |
| D63 | 113.1 | 8.16 | G114 | 109.1 | 8.34 |
| E64 | 120.3 | 7.24 | D115 | 117.9 | 7.9 |
| R65 | 116.4 | 8.05 | G116 | 109.2 | 8.01 |
| F66 | 120 | 8.99 | M117 | 114 | 8.24 |
| P67 |  |  | T118 | 111.7 | 8.98 |
| M68 | 122.3 | 8.5 | V119 | 121.1 | 8.15 |
| C69 | 124.4 | 11.4 | A120 | 121.3 | 9.09 |
| S70 |  |  | E121 | 117.9 | 7.71 |
| T71 | 112.6 | 7.75 | L122 | 121.9 | 8.87 |
| F72 | 117.1 | 7.7 | C123 | 119.6 | 8.27 |
| K73 | 122.1 | 7.49 | E124 | 119.1 | 7.66 |
| A74 | 116.8 | 6.68 | A125 | 123.9 | 8.21 |
| L75 | 113.5 | 6.65 | A126 | 120.2 | 8.41 |
| L76 | 118.4 | 7.88 | I127 | 112.1 | 7.75 |
| A77 | 117.3 | 7.48 | T128 | 106.4 | 8.39 |

| L129 | 117 | 6.64 | M186 | 115.6 | 8.43 |
| --- | --- | --- | --- | --- | --- |
| S130 | 117.2 | 7.1 | A187 | 119.8 | 7.74 |
| D131 | 121.9 | 7.32 | A188 | 119.9 | 7.43 |
| N132 | 126.8 | 8.82 | T189 | 120.7 | 8.92 |
| T133 | 121.1 | 7.7 | L190 | 121.1 | 8.68 |
| A134 | 122.6 | 8.88 | R191 | 118.7 | 8.03 |
| A135 | 114.7 | 7 | T192 | 117.8 | 8.2 |
| N136 | 119 | 7.8 | L193 | 116.9 | 8.1 |
| L137 | 121.4 | 8.83 | L194 | 113.9 | 8.41 |
| L138 | 119.8 | 7.89 | L195 | 114.6 | 7.72 |
| L139 | 120.8 | 8.76 | G196 | 110.3 | 7.36 |
| E140 | 122.2 | 8.78 | D197 | 118.8 | 8.34 |
| A141 | 122.1 | 7.69 | A198 | 121.8 | 7 |
| L142 | 115.9 | 7.7 | L199 | 117 | 9.09 |
| G143 | 102 | 7.5 | S200 | 118.4 | 9.29 |
| G144 | 109.3 | 8.43 | P201 |  |  |
| P145 |  |  | A202 | 118.8 | 8.34 |
| A146 | 118.9 | 8.58 | S203 | 119.5 | 7.93 |
| A147 | 121.7 | 7.25 | R204 | 123.1 | 9.09 |
| L148 | 120.9 | 7.45 | Q205 | 116.2 | 7.63 |
| T149 | 117.4 | 8.25 | Q206 | 118.1 | 7.43 |
| A150 | 122 | 8.04 | L207 | 120.5 | 7.8 |
| F151 | 120.6 | 7.78 | V208 | 118.1 | 8.04 |
| L152 | 121.5 | 8 | D209 | 118.9 | 8.48 |
| R153 | 118 | 8.26 | W210 | 120.4 | 7.94 |
| S154 | 116.8 | 7.85 | L211 | 117.5 | 8.03 |
| I155 | 113.2 | 6.97 | V212 | 122.8 | 9.22 |
| G156 | 108.3 | 7.41 | A213 | 122.1 | 7.5 |
| D157 | 121.9 | 7.83 | N214 | 116.2 | 7.28 |
| E158 | 123.8 | 8.53 | K215 | 125.9 | 9.03 |
| V159 | 121.1 | 8.81 | T216 | 108.5 | 7.59 |
| T160 | 126 | 9.86 | G217 | 112.6 | 9.05 |
| R161 | 120.9 | 8.7 | D218 | 123 | 8.77 |
| L162 | 121.4 | 7.5 | K219 | 113.8 | 8.36 |
| D163 | 126.9 | 9.94 | R220 | 117.9 | 6.87 |
| R164 | 124.8 | 9.19 | L221 | 125 | 7.84 |
| W165 | 113.9 | 7.66 | R222 | 113.4 | 9.06 |
| E166 | 116.9 | 9.32 | A223 | 118.4 | 7.14 |
| P167 |  |  | G224 | 105.3 | 7.96 |
| E168 | 128 | 8.54 | L225 | 118.1 | 6.64 |
| L169 | 113.5 | 7.72 | P226 |  |  |
| N170 | 119.1 | 7.55 | A227 | 123.6 | 8.46 |
| E171 | 122.4 | 7.69 | D228 | 111.3 | 8.47 |
| A172 | 117.7 | 9.46 | W229 | 122.3 | 7.72 |
| A173 | 120.9 | 8.39 | R230 | 125.3 | 7.77 |
| P174 |  |  | V231 | 124.6 | 8.84 |
| G175 | 113 | 8.79 | G232 | 111.5 | 9.07 |
| D176 | 122.5 | 7.22 | D233 | 122.6 | 8.18 |
| P177 |  |  | K234 | 113.3 | 8.43 |
| R178 | 123 | 7.83 | T235 | 114.1 | 6.7 |
| D179 | 115.6 | 8.91 | G236 | 103.1 | 7.65 |
| T180 | 105 | 7.2 | T237 |  |  |
| T181 | 113.8 | 8.09 | G238 | 111.9 | 8.36 |
| T182 | 111.3 | 8.47 | G240 | 105.7 | 8.18 |
| P183 |  |  | H241 | 112.8 | 8.75 |
| A184 | 117 | 9 | G242 | 103.8 | 8.54 |
| A185 | 124.1 | 8.27 | T243 | 114.9 | 6.76 |

| T244 | 126.7 | 9.27 | S268 | 109 | 7.45 |
| --- | --- | --- | --- | --- | --- |
| N245 | 118.4 | 9.01 | Q269 | 123.3 | 8.24 |
| D246 | 122.2 | 9.34 | V270 | 113.2 | 7.26 |
| I247 | 118.2 | 8.05 | D271 | 119.5 | 8.14 |
| A248 | 123.1 | 9.43 | A272 | 120.8 | 8.41 |
| V249 | 117.8 | 8.45 | D273 | 117.4 | 8.23 |
| I250 | 124.4 | 8.6 | A274 | 125.6 | 7.95 |
| W251 | 127.7 | 9.58 | R275 | 119.5 | 8.32 |
| P252 |  |  | D276 | 119.3 | 7.66 |
| P254 |  |  | A277 | 118.7 | 7.46 |
| G255 | 112.1 | 8.64 | V278 | 120.5 | 7.56 |
| R256 | 118.6 | 7.39 | I279 | 117.4 | 7.23 |
| A257 | 123 | 7.98 | A280 | 121.8 | 8.15 |
| P258 |  |  | E281 | 118 | 7.75 |
| I259 | 120.1 | 8.9 | V282 | 119.9 | 7.83 |
| V260 | 125.2 | 8.81 | G283 | 105.1 | 7.6 |
| V261 | 125.7 | 8.88 | R284 | 120.2 | 7.65 |
| T262 | 117.4 | 9.58 | L285 | 119.5 | 8.14 |
| V263 | 120.4 | 7.94 | V286 | 119.3 | 8.16 |
| Y264 | 127.3 | 9.28 | V287 | 118.2 | 7.49 |
| L265 | 124.2 | 7.89 | E288 | 115.6 | 7.46 |
| T266 | 118.8 | 9.17 | A289 | 120.2 | 7.9 |
| E267 | 119.5 | 8.39 | F290 | 123.7 | 7.72 |
